# Not a Simple Tether: Binding of *Toxoplasma gondii* AMA1 to RON2 during Invasion Protects AMA1 from Rhomboid-Mediated Cleavage and Leads to Dephosphorylation of Its Cytosolic Tail

**DOI:** 10.1128/mBio.00754-16

**Published:** 2016-09-13

**Authors:** Shruthi Krishnamurthy, Bin Deng, Roxana del Rio, Kerry R. Buchholz, Moritz Treeck, Siniša Urban, John Boothroyd, Ying-Wai Lam, Gary E. Ward

**Affiliations:** a Department of Microbiology and Molecular Genetics, University of Vermont, Burlington, Vermont, USA; b Department of Biology, University of Vermont, Burlington, Vermont, USA; c Department of Medicine, University of Vermont, Burlington, Vermont, USA; d Vermont Genetics Network Proteomics Facility, University of Vermont, Burlington, Vermont, USA; e Department of Microbiology and Immunology, Stanford School of Medicine, Stanford, California, USA; f Department of Molecular Biology and Genetics, Johns Hopkins University School of Medicine, Baltimore, Maryland, USA

## Abstract

Apical membrane antigen 1 (AMA1) is a receptor protein on the surface of *Toxoplasma gondii* that plays a critical role in host cell invasion. The ligand to which *T*. *gondii* AMA1 (TgAMA1) binds, TgRON2, is secreted into the host cell membrane by the parasite during the early stages of invasion. The TgAMA1-TgRON2 complex forms the core of the “moving junction,” a ring-shaped zone of tight contact between the parasite and host cell membranes, through which the parasite pushes itself during invasion. Paradoxically, the parasite also expresses rhomboid proteases that constitutively cleave the TgAMA1 transmembrane domain. How can TgAMA1 function effectively in host cell binding if its extracellular domain is constantly shed from the parasite surface? We show here that when TgAMA1 binds the domain 3 (D3) peptide of TgRON2, its susceptibility to cleavage by rhomboid protease(s) is greatly reduced. This likely serves to maintain parasite-host cell binding at the moving junction, a hypothesis supported by data showing that parasites expressing a hypercleavable version of TgAMA1 invade less efficiently than wild-type parasites do. Treatment of parasites with the D3 peptide was also found to reduce phosphorylation of S527 on the cytoplasmic tail of TgAMA1, and parasites expressing a phosphomimetic S527D allele of TgAMA1 showed an invasion defect. Taken together, these data suggest that TgAMA1-TgRON2 interaction at the moving junction protects TgAMA1 molecules that are actively engaged in host cell penetration from rhomboid-mediated cleavage and generates an outside-in signal that leads to dephosphorylation of the TgAMA1 cytosolic tail. Both of these effects are required for maximally efficient host cell invasion.

## IMPORTANCE

Nearly one-third of the world’s population is infected with the protozoan parasite *Toxoplasma gondii*, which causes life-threatening disease in neonates and immunocompromised individuals. *T. gondii* is a member of the phylum Apicomplexa, which includes many other parasites of veterinary and medical importance, such as those that cause coccidiosis, babesiosis, and malaria. Apicomplexan parasites grow within their hosts through repeated cycles of host cell invasion, parasite replication, and host cell lysis. Parasites that cannot invade host cells cannot survive or cause disease. AMA1 is a highly conserved protein on the surface of apicomplexan parasites that is known to be important for invasion, and the work presented here reveals new and unexpected insights into AMA1 function. A more complete understanding of the role of AMA1 in invasion may ultimately contribute to the development of new chemotherapeutics designed to disrupt AMA1 function and invasion-related signaling in this important group of human pathogens.

## INTRODUCTION

*Toxoplasma gondii* is a widespread protozoan parasite that infects up to 80% of the population in some regions of the world and causes life-threatening disease during pregnancy and in immunocompromised individuals (1, 2). As an obligate intracellular parasite, its ability to attach to and invade cells of its hosts is critical to its life cycle. Like other apicomplexan parasites, the invasive asexual form of *T. gondii*, the tachyzoite, contains three unique sets of secretory organelles, micronemes, rhoptries, and dense granules, which play distinct roles during and after host cell invasion (3). Micronemes are located at the apical end of the parasite and contain proteins involved in host cell recognition and attachment. The contents of the micronemes are secreted constitutively, and secretion increases during parasite interaction with host cells (4, 5). Secretion can also be induced experimentally by elevating parasite intracellular calcium levels using calcium ionophores (6, 7).

Apical membrane antigen 1 (AMA1) is a conserved, type I transmembrane protein of apicomplexan parasites that is secreted from the micronemes onto the parasite surface, where it functions in attachment to the host cell during invasion (8–13). The link between AMA1 and invasion was first suggested when antibodies against AMA1 were found to block invasion (10, 13, 14). Subsequently, parasites either conditionally depleted of AMA1 (12) or completely lacking AMA1 (8) showed reduced host cell attachment and a severe invasion defect. AMA1 localizes to the “moving junction,” a ring-like zone of tight apposition between the parasite and host cell plasma membranes, through which an invading parasite squeezes as it pushes its way into the host cell (15–18). At the moving junction, the extracellular domain of AMA1 physically connects the host cell and parasite membranes by binding to RON2, a protein secreted from the rhoptries into the host cell membrane during invasion (15, 19–22). Several independent studies identified domain 3 (D3) of RON2 as the region that binds to AMA1 with high (6 nM) affinity (16, 23–26). RON2, in turn, binds to other rhoptry-derived proteins (RON4, -5, and -8) secreted into the host cell (15, 21, 22, 27–30). Binding of this heterooligomeric RON protein complex to the host cell cytoskeleton would create the “anchor” against which the parasite exerts force during internalization (30–33).

This widely accepted model for the role of AMA1 at the moving junction was questioned recently by the observation that the small number of *ama1* knockout parasites that are able to invade do so with normal kinetics, suggesting that AMA1 functions early in invasion, i.e., in host cell attachment, but is not required at the moving junction for parasite internalization (8). However, it was subsequently shown that the parasite can make use of functional homologs of AMA1 to partially compensate for the loss of AMA1 (17). Despite this functional redundancy, parasites lacking AMA1 are completely avirulent in immunocompetent mice (34), reinforcing the importance of AMA1 in the parasite’s lytic cycle.

Like other microneme proteins, after *T. gondii* AMA1 (TgAMA1) has been trafficked onto the parasite surface, its transmembrane domain is cleaved, and its ectodomain is shed from the parasite (9, 10, 35–37). The intramembrane proteolysis of microneme proteins is mediated by rhomboid proteases, primarily *T. gondii* ROM4 (TgROM4), which is distributed over the entire parasite surface, and to a lesser extent TgROM5, which is also localized on the parasite surface but is concentrated at the posterior end (35–40). The function of microneme protein shedding is not entirely clear. It was recently proposed that shedding facilitates the establishment of an anterior-to-posterior concentration gradient of intact micronemal adhesins on the parasite surface (since new adhesins are continually secreted from the micronemes at the anterior end of the parasite), helping the parasite to orient properly during invasion (37). Whatever its function, constitutive intramembrane cleavage presents a problem for the model described above, since AMA1 cannot serve as an effective tether to the host cell if it is constantly being cleaved and shed from the parasite surface. The data presented here resolve this conundrum. We show that binding of TgAMA1 to TgRON2 significantly reduces its cleavage; thus, the subset of TgAMA1 molecules on the parasite surface that is actively engaged in host cell penetration is protected from rhomboid-mediated cleavage, stabilizing the interaction between the two cells. Furthermore, we report that binding of TgAMA1 to TgRON2 leads to the dephosphorylation of the TgAMA1 cytosolic tail (C-tail), suggesting that TgAMA1 plays a direct role in outside-in signaling during host cell invasion.

## RESULTS

### Treatment of parasites with the D3 peptide of TgRON2 reduces the amount of TgAMA1 shed from the parasite surface.

**FIG 1:**
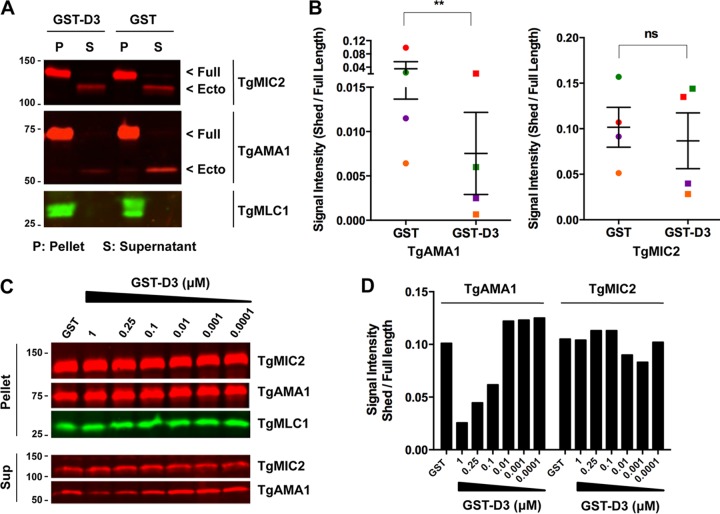
Treatment of parasites with GST-D3 peptide causes a dose-dependent reduction in the shedding of TgAMA1, but not TgMIC2. (A) Western blots from a microneme secretion assay using ARAMA1^WT^ parasites treated with either 5 µM GST-D3 or GST and probed with anti-TgMIC2, anti-FLAG (TgAMA1), and anti-TgMLC1. Assay pellet (P) and assay supernatant (S) fractions are indicated. Arrowheads to the right of the blots indicate the positions of full-length (Full) TgAMA1 or TgMIC2 and the corresponding shed ectodomains (Ecto). The numbers to the left of the blots indicate molecular masses (in kilodaltons). The doublet in the TgMLC1 blot likely represents different phospho forms of TgMLC1 (57). (B) Signal intensity ratios of ectodomain in the supernatant to full-length protein in the pellet from four independent microneme secretion assays were plotted for TgAMA1 and TgMIC2. Paired signal intensity values from each of the biological replicates were plotted using the same color symbols. Values are means ± standard errors of the means (SEM) (error bars). A paired one-tailed *t* test revealed a significant decrease in the amount of TgAMA1 shed from parasites treated with GST-D3 (**, *P* = 0.0018) but no corresponding decrease in the shedding of TgMIC2 (*P* = 0.1379; not significant [ns]). (C) Representative Western blots of microneme secretion assay using ARAMA1^WT^ parasites treated with 1 µM GST and six serial dilutions of GST-D3 (1 µM to 0.0001 µM). The pellet and supernatant (Sup) were each probed with anti-TgMIC2, anti-FLAG (TgAMA1), and anti-TgMLC1. GST-D3 caused a dose-dependent decrease in the amount of TgAMA1 (but not TgMIC2) ectodomain released into the assay supernatant. Numbers to the left of the blots indicate molecular masses (in kilodaltons). (D) Quantification of the Western blot in panel C. The signal intensity ratio of shed ectodomain in the supernatant to full-length protein in the pellet was plotted for both TgAMA1 and TgMIC2.

Parasites expressing FLAG-tagged TgAMA1 were generated by allelic replacement at the endogenous *TgAMA1* locus (see Fig. S1 in the supplemental material). The tag was placed within the extracellular domain (ectodomain) of TgAMA1 at a position that does not disrupt its function (35). These ARAMA1^WT^ parasites were pretreated either with glutathione *S*-transferase (GST) alone or with the D3 domain of RON2 fused to glutathione *S*-transferase (GST-D3) and used in constitutive microneme secretion assays. Parasites treated with 5 µM GST-D3 showed, on average, a 5.7-fold decrease in the amount of FLAG-tagged TgAMA1 ectodomain shed into the assay supernatant compared to GST-treated controls (Fig. 1A and B). The effect was dose dependent, increasing steadily from 0.01 to 1 µM GST-D3 (Fig. 1C and D). In contrast, GST-D3 treatment caused no significant decrease in the shedding of another microneme protein, TgMIC2, at concentrations as high as 5 µM (Fig. 1A to D). Similar results were observed when microneme secretion was induced with calcium ionophore: treatment of parasites with GST-D3, but not GST, resulted in a decrease in TgAMA1 ectodomain shedding with little or no effect on the shedding of TgMIC2 (see Fig. S2 in the supplemental material).

To control for any artifactual effects of the GST fusion, we also tested the effect of TgRON2-2, a cysteine-dicyclized synthetic peptide that encompasses the TgAMA1-binding residues within the TgRON2 D3 domain (23). Like GST-D3, treatment with TgRON2-2 resulted in a dose-dependent decrease in the amount of TgAMA1 ectodomain shed into the secretion assay supernatant (see Fig. S3A to S3D in the supplemental material), and TgRON2-2 concentrations as high as 5 µM had no effect on the shedding of either TgMIC2 (Fig. S3A, S3C, and S3D) or another microneme protein, TgMIC8 (Fig. S4).

### Treatment of parasites with GST-D3 inhibits the cleavage of TgAMA1, not its trafficking to the parasite surface.

**FIG 2:**
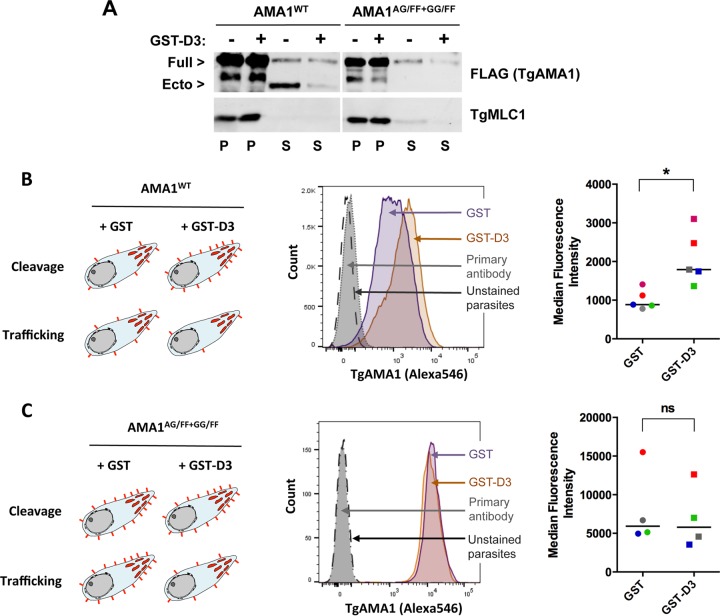
GST-D3 causes reduced cleavage of TgAMA1 but not reduced trafficking to the parasite surface. (A) A microneme secretion assay was performed using conditional AMA1^WT^ and AMA1^AG/FF+GG/FF^ parasites treated with either 1 µM GST-D3 (+) or GST (−). Assay pellet (P) and supernatant (S) fractions are indicated. The decrease in shedding of TgAMA1 was evident in AMA1^WT^ parasites treated with GST-D3 (compare AMA1^WT^ supernatants with and without GST-D3). No shed ectodomain was detected using either GST- or GST-D3-treated TgAMA1^AG/FF+GG/FF^ parasites (TgAMA1^AG/FF+GG/FF^ supernatants with and without GST-D3). Full-length protein in the supernatant is likely due to parasite lysis. TgMLC1 was used as a loading control. (B, left) Schematic showing the two possible outcomes from treatment of AMA1^WT^ parasites with GST-D3. If GST-D3 reduces cleavage of TgAMA1, we would expect more surface TgAMA1 (red spikes) after GST-D3 treatment than after GST treatment. If GST-D3 affects trafficking of TgAMA1 to the parasite surface, we would expect less TgAMA1 on the surface after GST-D3 treatment. (Middle) By flow cytometry, GST-D3 treatment resulted in an increase in surface TgAMA1 in AMA1^WT^ parasites, consistent with an effect on cleavage. Representative histograms are shown. The dashed-line histogram represents unstained parasites, the gray histogram represents parasites stained with primary antibody only, the purple histogram represents parasites treated with GST, and the orange histogram represents parasites treated with GST-D3. Alexa546 indicates Alexa Fluor 546 fluorescence. (Right) Combined flow data from five biological replicates. The bars indicate the median values of the TgAMA1 signals. Paired samples from each experiment are indicated using symbols of the same color. The median values were significantly different (*, *P* = 0.0159) by a nonparametric two-tailed *t* test. (C, left) If GST-D3 inhibits cleavage, there would likely be no difference in the amount of TgAMA1 on the surface of GST-treated versus GST-D3-treated parasites expressing noncleavable TgAMA1 (AMA1^AG/FF+GG/FF^), since cleavage is already severely inhibited in these mutant parasites (see panel A). On the other hand, if GST-D3 affects trafficking of TgAMA1 to the parasite surface, we would expect less TgAMA1 on the surface of AMA1^AG/FF+GG/FF^ parasites following treatment with GST-D3. (Middle and right) Flow cytometry (performed and analyzed as in panel B) showed that GST-D3 treatment had no significant (ns) effect (*P* = 0.6571) on the amount of AMA1^AG/FF+GG/FF^ on the parasite surface, again consistent with an effect on cleavage rather than trafficking.

The reduced amount of TgAMA1 ectodomain recovered in the assay supernatant after GST-D3 treatment (shown again in Fig. 2A, third and fourth lanes) could be due to either reduced trafficking of full-length TgAMA1 from the micronemes onto the parasite surface or reduced intramembrane cleavage of TgAMA1 once it reaches the surface. If GST-D3 inhibits trafficking, we would expect to find less TgAMA1 on the parasite surface, but if it inhibits cleavage, we would expect to find more (Fig. 2B, left panel). To discriminate between these possibilities, we made use of parasites that contain an anhydrotetracycline (ATc)-repressible copy of wild-type TgAMA1 and a second, FLAG-tagged copy of TgAMA1 that is either wild type (AMA1^WT^) or contains mutations within its transmembrane domain (AMA1^AG/FF+GG/FF^) that render it resistant to cleavage by rhomboid proteases (35). Following ATc treatment, AMA1^WT^ parasites (which express the FLAG-tagged wild-type allele of TgAMA1) were treated with either GST or GST-D3, and the amount of TgAMA1 on the surface of the parasites was quantified by flow cytometry (37, 39). There was a significant increase in the amount of TgAMA1 on the surface of GST-D3-treated parasites compared to parasites treated with GST alone (Fig. 2B, middle and right panels). Similar to the Western blot-based measurements of microneme secretion, the effect of GST-D3 on TgAMA1 surface abundance showed a clear dose dependence by flow cytometry (see Fig. S5 in the supplemental material). For a control, we measured the amount of glycosylphosphatidylinositol (GPI)-anchored TgSAG1 on the parasite surface (39) and found it to be unaffected by GST-D3 treatment (see Fig. S6 in the supplemental material).

These data are consistent with GST-D3 inhibiting surface cleavage of TgAMA1, rather than its trafficking from the micronemes. To independently confirm this, we did a similar experiment using the AMA1^AG/FF+GG/FF^ parasites, which, after ATc treatment, express the cleavage-resistant TgAMA1 allele (Fig. 2A, AMA1^AG/FF+GG/FF^). In these mutant parasites, we would expect less TgAMA1 on the surface if GST-D3 reduces its trafficking, but little or no difference in the amount of TgAMA1 on the surface if GST-D3 treatment inhibits cleavage, since cleavage is already low in the mutant (Fig. 2C, left panel). We observed the latter result: no difference in the amount of AMA1^AG/FF+GG/FF^ was detected on the parasite surface after treatment with GST-D3 (Fig. 2C, middle and right panels). Taken together, these data argue that the binding of TgRON2 to TgAMA1 reduces TgAMA1 intramembrane cleavage.

**FIG 3:**
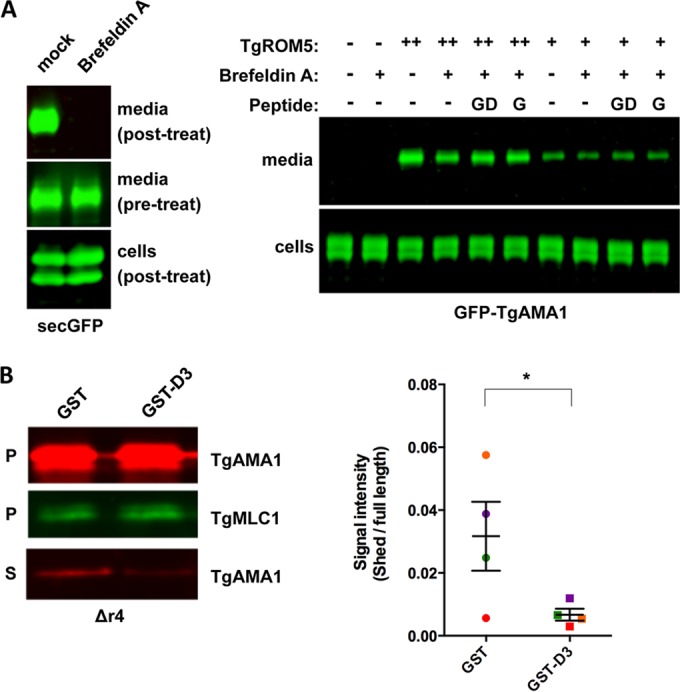
TgAMA1 cleavage by TgROM5 is inhibited by GST-D3 treatment in parasites but not in mammalian cells. (A, left) HEK293 cells were transfected with a secreted GFP construct (secGFP) to monitor the efficiency of blocking secretion with 10 µM brefeldin A relative to mock-treated cells. While cells efficiently secreted GFP prior to brefeldin A treatment (middle panel), brefeldin A blocked all GFP secretion (top panel). (Right) HEK293 cells were cotransfected with GFP-TgAMA1 and high (++) or low (+) levels of TgROM5. The cells were then incubated for 2.5 h in medium containing 10 µM brefeldin A (+) or lacking (−) brefeldin A (to block release of product resulting from any potential intracellular cleavage) with no peptide (−) or with 5 µM GST (G) or 5 µM GST-D3 (GD). Anti-GFP Western blots show cleaved GFP-TgAMA1 ectodomain released into the culture supernatant. (B, left) Western blots from a microneme secretion assay of Δ*r4* parasites, which lack TgROM4 (37). Parasites were pretreated with either 1 µM GST or GST-D3, as indicated. Assay pellet (P) and supernatant (S) fractions are indicated. TgMLC1 was used as a loading control. (Right) Quantification of the results from four independent microneme assays, showing signal intensity ratio of TgAMA1 ectodomain in the supernatant to full-length protein in the pellet. Paired signal intensity values from each of the biological replicates are indicated by the same color symbols. Values are means ± SEM (error bars). GST-D3 treatment reduces TgAMA1 shedding in the Δ*r4* parasites, based on a paired one-tailed *t* test (*, *P* = 0.0159).

To test whether the binding of TgRON2 inhibits rhomboid-mediated cleavage of TgAMA1 directly, we co-expressed TgAMA1 and the rhomboid protease TgROM5 in mammalian HEK293 cells and measured the amount of TgAMA1 released into the culture supernatant in the presence or absence of GST-D3. We used brefeldin A in these experiments to block secretion of any potential intracellularly processed TgAMA1 (Fig. 3A, left blot). GST-D3 bound to the surface of the TgAMA1-expressing cells (data not shown) but had no detected effect on the amount of TgAMA1 shed (Fig. 3A, right blot). Although TgROM4 is the protease responsible for the majority of TgAMA1 cleavage in the parasite (36, 37), no one has yet succeeded in expressing functional TgROM4 in mammalian cells (38, 40). However, TgROM5 is responsible for most of the residual TgAMA1 cleavage in parasites lacking TgROM4 (36, 37), and we found that GST-D3 treatment inhibits this TgROM5-mediated cleavage of TgAMA1 (Fig. 3B). Thus, GST-D3 inhibits rhomboid-mediated TgAMA1 cleavage in parasites but not mammalian cells, suggesting the involvement of additional parasite-specific factors in the underlying mechanism (see Discussion).

### Functional consequences of altered TgAMA1 cleavage.

**FIG 4:**
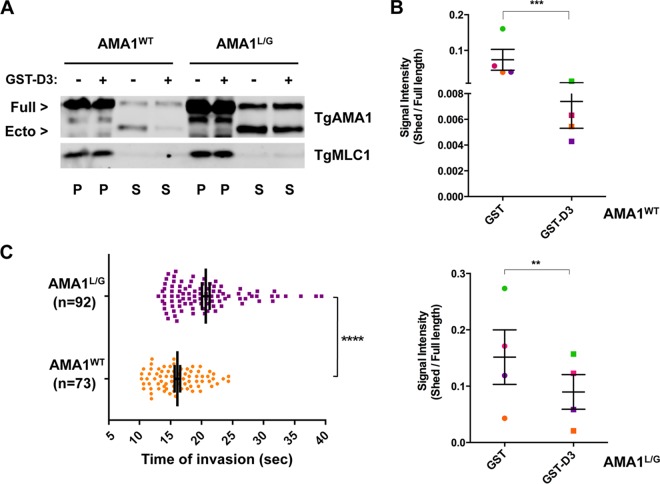
Parasites expressing a hypercleavable form of TgAMA1 (AMA1^L/G^) take longer to invade than parasites expressing AMA1^WT^. (A) A microneme secretion assay was performed using conditional AMA1^WT^ and AMA1^L/G^ parasites treated with either 1 µM GST-D3 (+) or GST (−). GST-D3 treatment caused decreased shedding of TgAMA1 in both parasite lines, but there was considerable cleavage and shedding of the mutant protein even after GST-D3 treatment. TgMLC1 was used as a loading control. Full-length TgAMA1 detected in the supernatants (S) is likely due to parasite lysis. P, pellet. (B) Signal intensity ratios of ectodomain in the supernatant to full-length protein in the pellet from four independent microneme secretion assays using AMA1^WT^ and AMA1^L/G^ parasites treated with either 1 µM GST or GST-D3. Paired signal intensity values from each of the biological replicates were plotted using the same color symbols. Values are means ± SEM (error bars). A paired one-tailed *t* test revealed a significant decrease in the amount of TgAMA1 shed from both AMA1^WT^ parasites (***, *P* = 0.0001) and AMA1^L/G^ parasites (**, *P* = 0.0039) following treatment with GST-D3. (C) Quantification of the duration (in seconds) of host cell penetration by AMA1^L/G^ (*n* = 92) and AMA1^WT^ (*n* = 73) parasites. Mean penetration times, indicated by tall vertical lines, were 20.7 and 16.1 s, respectively. Shorter vertical lines indicate SEM. A two-tailed unpaired *t* test with Welch’s correlation revealed a significant difference between the two data sets (****, *P* < 0.0001).

If interaction of TgAMA1 with TgRON2 at the moving junction inhibits cleavage of TgAMA1, this could stabilize the junction and enable efficient parasite penetration into the host cell. To test this hypothesis, we used a parasite line expressing a hypercleavable mutant form of TgAMA1 (AMA1^L/G^), which shows a >20-fold increase (35) in constitutive cleavage compared to AMA1^WT^ (Fig. 4A, compare third and seventh lanes). Treatment of the AMA1^L/G^ parasites with saturating amounts of GST-D3 inhibits this cleavage by ~50% (Fig. 4A seventh and eighth lanes, and Fig. 4B). However, because the baseline cleavage of the mutant protein is so high, significantly more shedding of mutant TgAMA1 is observed in the presence of GST-D3 than is seen with wild-type TgAMA1 in AMA^WT^ parasites (Fig. 4A, compare fourth and eight lanes).

Despite this increase in TgAMA1 shedding, the AMA1^L/G^ parasites are capable of normal levels of invasion in a 60-min endpoint assay (35) and show no obvious difference in the moving junction compared to that seen in invading AMA1^WT^ parasites (data not shown). However, when live invasion assays were performed to compare the kinetics of invasion, AMA1^WT^ parasites took on average 16.1 s to internalize (range, 10.33 to 24.34 s), whereas the AMA1^L/G^ parasites took longer, on average 20.7 s (range, 13.04 to 39.36 s; Fig. 4C).

Taken together, these data suggest a model in which the binding of TgRON2 to TgAMA1 at the moving junction protects the TgAMA1 molecules that are actively engaged in host cell penetration from rhomboid-mediated cleavage, enabling maximally efficient host cell invasion.

### Interaction of TgAMA1 with TgRON2 reduces phosphorylation on the C-tail of TgAMA1.

**FIG 5:**
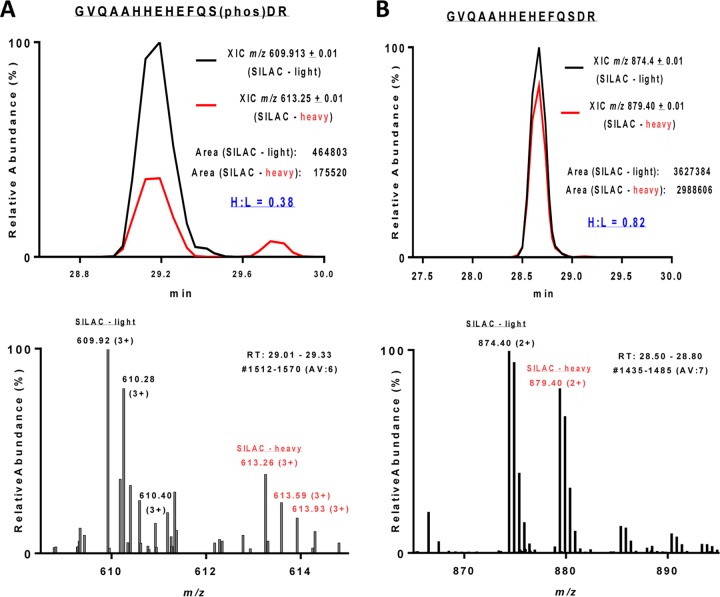
Treatment of parasites with GST-D3 results in dephosphorylation of S527 on the C-tail of TgAMA1. The extracted ion chromatograms (XIC) of light- and heavy-labeled TgAMA1 peptides eluting symmetrically at the same retention time (RT) are shown in the top panels, and the corresponding MS1 spectra of the light and heavy isotopologues are shown in the bottom panels. The SILAC heavy/light (H/L) ratio was quantified by precursor ion elution profiles. (A) The abundance of the phosphopeptide GVQAAHHEHEFQS(phos)DR (phos stands for phosphorylated) was decreased in the heavy-labeled (GST-D3-treated) parasites, with an H/L ratio of 0.38. (B) The nonphosphorylated TgAMA1 S527-containing peptide was relatively unchanged between light-labeled (GST-treated) and heavy-labeled (GST-D3-treated) parasites, with an H/L ratio of 0.82. The mean H/L ratio of the other TgAMA1 peptides in this experiment was 0.76 ± 0.21. AV, average.

Given that binding of GST-D3 to the extracellular domain (23, 26) of TgAMA1 affects cleavage of residues within the TgAMA1 transmembrane domain, we asked whether the binding might have other distal effects, altering some aspect of TgAMA1 C-tail function. Phosphorylation of S610 on the C-tail of *Plasmodium falciparum* AMA1 (PfAMA1) is known to be important for invasion (41, 42). The C-tail of TgAMA1 was also recently shown to be phosphorylated, but on a different residue, S527 (43). To test whether TgRON2 binding to TgAMA1 affects the phosphorylation state of S527, we metabolically labeled parasites by stable isotope labeling with amino acids in cell culture (SILAC) (44, 45) with either “heavy” or “light” isotopic versions of l-arginine and l-lysine. The heavy-labeled parasites were then treated with GST-D3, and the light-labeled parasites were treated with GST. TgAMA1 was immunoprecipitated from each sample, and mass spectrometry was used to quantify the heavy and light tryptic peptides recovered. The relative heavy/light (H/L) ratio of the phosphorylated S527-containing peptide in the two samples was compared to the H/L ratio of all TgAMA1 tryptic peptides recovered. The analysis revealed a consistent 30 to 40% reduction in S527 phosphorylation following treatment with GST-D3 (Fig. 5; see Fig. S7 and S8 in the supplemental material).

**FIG 6:**
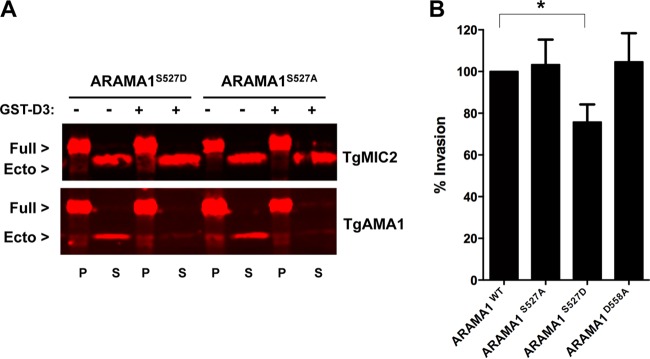
Parasites expressing the S527D phosphomimetic allele of *TgAMA1* invade host cells less efficiently than parasites expressing wild-type TgAMA1. (A) Western blots from a microneme secretion assay of ARAMA1^S527A^ and ARAMA1^S527D^ parasites, treated with either 1 µM GST-D3 (+) or GST (−). Pellet (P) fractions contain full-length (Full) TgAMA1 and TgMIC2, and the supernatant (S) fractions contain corresponding shed ectodomains (Ecto). (B) Laser scanning cytometry-based invasion assay comparing host cell invasion by the four allelic replacement parasite lines. Values are means plus SEM (error bars) from multiple independent biological replicates. The invasion level of ARAMA1^WT^ was set at 100% in each assay. The ARAMA1^S527D^ parasites showed 24.2% less invasion than the ARAMA1^WT^ parasites (*, *P* = 0.0159 by one-way analysis of variance [ANOVA] with uncorrected Fisher’s least-significant difference [LSD] test).

To test whether the phosphorylation state of S527 is functionally important, parasites expressing nonphosphorylatable (S527A) or phosphomimetic (S527D) forms of TgAMA1 were generated by allelic replacement. Diagnostic PCR confirmed integration of the mutant alleles at the endogenous *TgAMA1* locus, and the mutant proteins localized properly and were expressed at levels similar to those of ARAMA1^WT^ (see Fig. S9A to S9C in the supplemental material). The ARAMA1^S527A^ and ARAMA1^S527D^ parasite lines shed similar amounts of TgAMA1, and shedding was reduced in both lines following treatment with GST-D3 (Fig. 6A), demonstrating that GST-D3-induced dephosphorylation of S527 does not play a direct role in the GST-D3-induced reduction in TgAMA1 shedding. In invasion assays, parasites expressing the nonphosphorylatable S527A allele invaded host cells to levels indistinguishable from those of the wild-type parasites. In contrast, parasites expressing the phosphomimetic S527D allele showed a consistent 25% decrease in host cell invasion (Fig. 6B), suggesting that the TgRON2-induced dephosphorylation of TgAMA1 is necessary for optimal host cell invasion.

The site on PfAMA1 that needs to be phosphorylated for invasion to occur, Ser610, does not align with S527 in TgAMA1, but instead corresponds to a potentially phosphomimetic TgAMA1 residue, D558. To determine whether the negative charge on D558 is important for C-tail function and invasion in *T. gondii*, parasites expressing a D558A mutant allele of *TgAMA1* were generated by allelic replacement (see Fig. S9B and S9C in the supplemental material). These parasites showed no defect in invasion (Fig. 6B). This result, together with the observation that C-tail dephosphorylation rather than phosphorylation is required for optimal invasion in *T. gondii*, provides compelling evidence that AMA1 C-tail function is regulated differently in *T. gondii* and *P. falciparum*.

## DISCUSSION

During invasion, AMA1 (embedded in the parasite plasma membrane) binds with high affinity to RON2 (embedded in the host cell plasma membrane) to form the core of the moving junction. We show here that when TgAMA1 engages with TgRON2, it becomes less susceptible to rhomboid-mediated intramembrane cleavage. We therefore propose that TgAMA1 cleavage is regulated spatially on the parasite surface. Most of the surface TgAMA1 is constitutively cleaved and shed, establishing an anterior to posterior gradient of TgAMA1 that may be important for attachment or junction formation (37), but the TgAMA1 molecules that are actively engaged with TgRON2 at the moving junction are resistant to cleavage, enabling them to serve as effective tethers between the two cells. Consistent with this model, parasites expressing a mutant form of TgAMA1 in which the transmembrane domain is more readily cleaved by surface rhomboids showed a modest but significant delay in their invasion kinetics (Fig. 4C). Note that while TgAMA1 cleavage is greatly enhanced in these mutant parasites, it is still partially inhibited by GST-D3 (Fig. 4B), which may explain why the effect on invasion is not more pronounced, i.e., even though the mutant TgAMA1 is more susceptible to cleavage than the wild type, binding to TgRON2 still provides some degree of protection against cleavage and a proportion of the parasite-host cell tethers therefore likely remain intact.

The mechanism by which TgRON2 binding inhibits TgAMA1 cleavage is not known. GST-D3 clearly inhibits cleavage by TgROM4, since TgROM4 is responsible for the great majority of TgAMA1 cleavage in parasites (36, 37), and treatment with the peptide inhibits 80 to 90% of total TgAMA1 cleavage (Fig. 1; see Fig. S3 in the supplemental material). GST-D3 treatment also inhibits TgAMA1 cleavage by TgROM5 (Fig. 3B). However, the effect of the peptide on TgAMA1 cleavage is unlikely to be mediated through a general inhibition of rhomboid protease activity, since treatment with GST-D3 did not alter the processing of TgMIC2 and TgMIC8 (Fig. 1; see Fig. S4 in the supplemental material), which are cleaved by the same proteases (36, 37). Binding of TgRON2 to TgAMA1 is known to induce a conformational change in the TgAMA1 ectodomain (23) that could somehow be propagated to the transmembrane domain, making it a less suitable substrate for rhomboid protease cleavage. This also seems unlikely, since we expressed TgAMA1 and TgROM5 in mammalian cells and saw no difference in the amount of TgAMA1 shed in the presence or absence of GST-D3 (Fig. 3A), whereas GST-D3 treatment did inhibit TgROM5-mediated shedding in parasites (Fig. 3B). Inhibition of rhomboid-mediated TgAMA1 cleavage by GST-D3 in parasites but not mammalian cells may reflect the involvement of an additional protein(s) that is present in the parasite but not the mammalian cell. Alternatively, the physicochemical environment within the parasite and mammalian cell plasma membranes may be different in some way that alters the dynamics of the TgAMA1 transmembrane helix and/or its propensity to enter into the rhomboid active site, affecting cleavage (46).

The phosphorylation of S610 on the C-tail of PfAMA1 is important for invasion of erythrocytes by *P. falciparum* merozoites (41, 42). Intriguingly, alignment of the orthologous AMA1 C-tails from 13 different apicomplexan parasites reveals that S610 is highly conserved across the phylum except in *T. gondii*, where it is replaced by potentially phosphomimetic D558 (19). *T. gondii* tachyzoites expressing TgAMA1 with a D558A mutation invaded cells normally (Fig. 6B), indicating that C-tail function is regulated via different mechanisms in *T. gondii* and *P. falciparum*. Indeed, the TgAMA1 C-tail is phosphorylated on a different residue, S527, and we show here that GST-D3 treatment of parasites results in a 30 to 40% decrease in S527 phosphorylation (Fig. 5; see Fig. S8 in the supplemental material). When wild-type TgAMA1 was replaced with a TgAMA1 S527D phosphomimetic allele, we found that invasion was partially inhibited (Fig. 6B). The effects of GST-D3 are very likely induced through direct binding to TgAMA1, since in the absence of TgAMA1, no detected peptide binds to the parasite surface (24). Taken together, these data suggest that when TgAMA1 binds to TgRON2, an outside-in signal is generated that leads to dephosphorylation of the AMA1 C-tail, and this dephosphorylation is required for maximally efficient invasion. The stoichiometry of phosphorylation on S527 appears to be low, based on the relative abundance of the phospho and dephospho forms of the S527-containing peptide (e.g., compare the areas of the extracted ion chromatograms in Fig. 5) and the observation that a decrease in the abundance of the phospho form following GST-D3 treatment was not associated with a corresponding increase in the abundance of the dephospho form (Fig. 5; see Fig. S8 in the supplemental material). Further studies will be required to determine whether the low stoichiometry reflects localized phosphorylation of a specific subset of TgAMA1 molecules within the parasite, e.g., those that have been trafficked from the micronemes onto the parasite surface. If this were the case, dephosphorylation of the TgAMA1 C-tail at the moving junction could serve to distinguish TgAMA1 molecules that are actively involved in invasion from the rest of the surface TgAMA1. It is worth noting that at least two protein phosphatases have been previously implicated in attachment and invasion, one of which localizes to the apical end of the parasite prior to invasion (47, 48).

In summary, our data show that the binding of the ectodomain of TgAMA1 to TgRON2 does more than just tether the parasite to the host cell. This interaction has two additional effects on TgAMA1, distal to the site of binding: it protects the transmembrane domain of TgAMA1 from cleavage by cell surface rhomboid protease(s), and it generates an outside-in signal that leads to dephosphorylation of the TgAMA1 C-tail, enhancing invasion efficiency. These effects may contribute to the ability of exogenously added GST-D3 to reduce the efficiency of host cell invasion by *T. gondii* (19). Inhibition was originally presumed to be due to simple competition between the added GST-D3 and native TgRON2 in the host cell plasma membrane for binding of TgAMA1, but the results presented here indicate that GST-D3 binding has additional important effects on TgAMA1 function. Future studies will focus on the mechanism of TgRON2-induced cleavage resistance and the role of TgAMA1 dephosphorylation in invasion and subsequent events of the parasite’s lytic cycle.

## MATERIALS AND METHODS

### Host cells and parasite culture.

Human foreskin fibroblasts (HFFs) (ATCC CRL-1643) were grown at 37°C with 5% CO_2_ and humidity in growth medium (Dulbecco’s modified Eagle’s medium [DMEM] supplemented with 10 mM HEPES [pH 7.2], 10 units/ml penicillin, and 10 units/ml streptomycin sulfate) containing 10% (vol/vol) fetal bovine serum (FBS). Wild-type and allelic replacement parasites were grown in HFFs maintained in growth medium with 1% FBS (49). TgAMA1 conditional knockdown (KD_i_) parasites (12) were maintained in growth medium with 1% FBS and 25 µg/ml mycophenolic acid, 50 µg/ml xanthine, 1 µM pyrimethamine, and 20 µM chloramphenicol. Thirty-six hours before experiments with the AMA1 KD_i_ parasites, infected monolayers were switched to the same medium containing 1.5 µg/ml anhydrotetracycline (ATc) (12). The Δ*r4* parasites were grown and maintained as described in reference 37.

Infected cells containing large intracellular vacuoles were detached from the flask using a cell scraper, and parasites were released by passage through a blunt 26-gauge needle. Host cell debris was removed using a sterile 3-µm Nuclepore (Whatman) filter. Unless otherwise indicated, parasites were centrifuged at 2,500 rpm at 25°C for 4 min and resuspended in medium prior to use.

### Generation of FLAG-tagged allelic replacement parasites.

Phleomycin-resistant parasites containing either wild-type or mutant FLAG-tagged TgAMA1 at the *TgAMA1* endogenous locus were generated using the vector pA/TgAMA1^WT^ Flag.BLE as described in reference 35 and outlined in Fig. S1 in the supplemental material. QuikChange mutagenesis was used to introduce the desired point mutation(s) into pA/TgAMA1^WT^ Flag.BLE using the primers listed in Table S1. Following parasite transfection and selection, single clones were screened for integration of *Flag-TgAMA1* at the correct locus using the primers listed in Table S1 (p1 to p4). The four allelic replacement parasite lines used in this study are designated ARAMA1^WT^, ARAMA1^527A^, ARAMA1^527D^, and ARAMA1^D558A^.

### Immunofluorescence analysis.

Confluent monolayers of HFFs on 25-mm circular glass coverslips were infected with parasites for 12 h, fixed for 15 min in phosphate-buffered saline (PBS) containing 2.5% (vol/vol) paraformaldehyde and permeabilized for 15 min in PBS containing 0.25% (vol/vol) Triton X-100 (TX-100). After the coverslips were blocked for 30 min in blocking buffer (PBS containing 1% [wt/vol] bovine serum albumin [BSA]), they were incubated for 15 min in blocking buffer containing mouse anti-FLAG (Sigma-Aldrich) or rabbit anti-TgIMC1 (50), each at a dilution of 1:1,000, followed by incubation for 15 min with Alexa Fluor 488-conjugated goat anti-mouse IgG (Invitrogen) or Alexa Fluor 546-conjugated goat anti-rabbit IgG (Invitrogen), each at a 1:1,000 dilution in blocking buffer. The coverslips were mounted on glass slides and imaged using the 100× objective of a Nikon Eclipse TE 300 epifluorescence microscope.

### Microneme secretion assays.

GST and GST-D3 were purified from *Escherichia coli* (Rosetta strain; Novagen/EMD Millipore) as previously described (24, 51) except that no detergent was used in the lysis or wash buffers. The purified proteins were dialyzed against 10 mM Tris-HCl (pH 8.0)−150 mM NaCl and concentrated in Amicon Ultra 0.5-ml 10,000 (10K) membrane centrifugal filters (Millipore). Glycerol was added to 20% (vol/vol), and the stocks were stored at −80°C.

Microneme secretion assays were performed as described previously (12) with minor modifications. Briefly, parasites were harvested, pelleted at 1,000 × *g* for 8 min at 4°C, and counted. A total of 2 × 10^8^ parasites were resuspended in 50 µl of microneme secretion medium (DMEM containing 10 mM HEPES [pH 7.2], 2% [wt/vol] ovalbumin, and 1% [vol/vol] FetalPlex [Gemini Bio-Products]). GST and GST-D3 stocks were diluted in Hanks buffered salt solution (HBSS), and 50 µl of this solution was added to the parasite suspension. To induce secretion, ionomycin was added to a final concentration of 1 µM. Parasites were incubated at 37°C with CO_2_ for 10 and 30 min for induced and constitutive secretion, respectively, placed on ice for 5 min, and centrifuged at 1,200 × *g* for 5 min. The pellet and supernatant fractions were analyzed by SDS-PAGE/Western blotting. Secretion assays with TgRON2-2 (generously provided by Marty Boulanger) were done in DMEM with 1% dialyzed FBS (Invitrogen) and 20 mM HEPES (pH 7.2), and the total volume of parasites and peptide was 120 µl (52). For secretion assays with AMA1 KD_i_ parasites (AMA1^WT^, AMA1^L/G^, and AMA1^AG/FF+GG/FF)^, the assay medium was HBSS with 100 mM HEPES (pH 7.2), and the parasites were incubated for 15 min at 37°C (35).

Immunoblots were probed with mouse anti-TgMIC2 (generous gift from Vern Carruthers; 1:10,000), mouse anti-FLAG (1:10,000), rabbit anti-TgMIC8 (generous gift from Markus Meissner; 1:500), and rabbit anti-TgMLC1 (1:1,000) in Odyssey LI-COR block buffer (LI-COR Biosciences), followed by incubation with IRDye 680-conjugated anti-rabbit IgG and IRDye 800-conjugated anti-mouse IgG (LI-COR), each at 1:20,000 in PBS containing 0.5% BSA. The blots were washed in PBS and scanned using an Odyssey CLx infrared imager (LI-COR). Images were processed using Image Studio software (LI-COR). After adjusting for parasite equivalents loaded (in all cases the percentage of the pellet fraction loaded was half that of the supernatant fraction), the signal intensity ratio of the band in each pair of pellet and supernatant fractions was calculated and plotted using GraphPad Prism 6.

### Flow cytometry.

Parasites were harvested and resuspended in motility buffer (1× minimal essential medium [MEM], 1% FBS, 10 mM GlutaMAX [Thermo Fisher], 10 mM HEPES [pH 7.2]) or microneme secretion medium with 1% ovalbumin and 0.5% FetalPlex. A total of 3 × 10^7^ parasites were incubated with either GST or GST-D3 at 37°C on a nutator for 30 min and prepared for flow cytometry (39). Briefly, parasites were fixed using 4% (vol/vol) paraformaldehyde in PBS for 20 min on ice, washed three times in blocking buffer (PBS containing 1% [wt/vol] BSA and 1% [vol/vol] goat serum) and incubated for 20 min in this buffer. Parasites were then incubated for 15 min with 5 µg/ml mouse anti-FLAG, 20 µg/ml fluorescein isothiocyanate (FITC)-conjugated rabbit anti-*T. gondii* (ab20907 [Abcam] [Western blotting revealed that >95% of the immunoreactivity of this antibody is directed against TgSAG1 {data not shown}]) or 2 µg/ml rabbit anti-GST (Immunology Consultants Laboratory [ICL] antibodies). After four washes at 1,000 × *g* for 2 min each, parasites were incubated with Alexa Fluor 546-conjugated goat anti-mouse IgG or Alexa Fluor 488-conjugated goat anti-rabbit IgG at a 1:500 dilution for 15 min. Parasites were washed four times and resuspended in 200 µl of blocking buffer. Flow cytometric analysis was performed on a MACSQuant VYB instrument (Miltenyi Biotech) equipped with 561-nm and 488-nm lasers. The Alexa Fluor 546 signal (by 561-nm laser) was detected using a 586/15 bandpass filter and Alexa Fluor 488/FITC signals (by 488-nm laser) using a 525/50 bandpass filter. Data were acquired using MACSQuantify v2.5 software and analyzed with FlowJo software (TreeStar v10).

### Live imaging of parasite invasion.

Parasite invasion kinetics were measured as described previously (35), except that the invasion medium was 1× MEM containing 1% FBS, 10 mM GlutaMAX, and 10 mM HEPES (pH 7.2).

### Mass spectrometry. (i) SILAC and immunoprecipitation.

SILAC DMEM medium (Thermo Fisher) was supplemented with 10% dialyzed FBS, 10 mM HEPES (pH 7.2), and 10 units/ml each of penicillin and streptomycin sulfate. Heavy SILAC medium also contained stable isotopic forms of “heavy” l-arginine-HCl (^13^C_6_, ^15^N_4_) at 0.398 mM and l-lysine-2HCl (^13^C_6_, ^15^N_2_) at 0.798 mM (Cambridge Isotope Laboratories). The light media contained naturally occurring “light” isotopic forms of l-arginine and l-lysine at 0.398 mM and 0.798 mM, respectively (53). l-Proline was added to the media to 40 mg/liter in order to prevent arginine-to-proline conversion (54). HFFs were grown in heavy or light SILAC medium for five or six passages. Two days prior to the experiment, 12 T75 flasks of heavy- and light-labeled host cells were infected with parasites that had been cultured in heavy- or light-labeled host cells for one complete lytic cycle.

Each set of 12 T75 flasks yielded approximately 7 × 10^8^ to 10 × 10^8^ of freshly harvested parasites, which were treated for 30 min at 37°C with a 5 µM concentration of either GST-D3 (heavy-labeled parasites) or GST (light-labeled parasites). After washing with cold PBS, parasite proteins were extracted on ice for 10 min in 1 ml of TX-100 lysis buffer (1% TX-100, 50 mM Tris-HCl [pH 8], 150 mM NaCl, 2 mM EDTA [kinase inhibitor], 1:200 protease inhibitor mix [catalog no. P8340; Sigma], and phosphatase inhibitors). Phosphatase inhibitor stocks, prepared separately in water and consisting of sodium orthovanadate at 100 mM, 1 M β-glycerophosphate, and 125 mM sodium pyruvate, were added to the lysis buffer to final concentrations of 0.1 mM, 1 mM, and 2.5 mM, respectively. Immunoprecipitation was performed using 0.0725 mg/ml anti-TgAMA1 antibody B3-90 (9), followed by incubation with 50 µl of recombinant protein A-Sepharose beads (Life Technologies). Bound proteins were eluted by boiling in 150 µl of 1× Laemmli sample buffer containing 5% (vol/vol) β-mercaptoethanol, resolved on a 12% SDS-polyacrylamide gel, and Coomassie blue stained.

### (ii) Trypsin digestion.

The band containing 75-kDa TgAMA1 was excised and subjected to reduction, alkylation, and in-gel trypsin digestion, as previously described (55).

### (iii) Liquid chromatography-mass spectrometry.

The tryptic digestion products were dissolved in 20 µl of 0.1% formic acid and 2.5% acetonitrile, and 6 µl was loaded onto a fused silica microcapillary liquid chromatography (LC) column (12 cm by 100 µm [inner diameter]) packed with C_18_ reversed-phase resin (5-µm particle size; 20-nm pore size; Magic C_18_AQ, Michrom Bioresources). Peptides were separated by applying a gradient of 3 to 45% acetonitrile in 0.1% formic acid at a flow rate of 500 nl/min for 75 min. Nanospray was used to introduce peptides into a linear ion trap LTQ Orbitrap mass spectrometer (Thermo Fisher) via a nanospray ionization source. Mass spectrometry (MS) data were acquired in a data-dependent acquisition mode, in which an Orbitrap survey scan from *m/z* 360 to 1,700 (resolution, 30,000 full width at half maximum [FWHM] at *m/z* 400) was paralleled by 10 LTQ tandem mass spectrometry (MS/MS) scans of the most abundant ions. The “lock mass” option was utilized in all full scans. After a liquid chromatography-mass spectrometry (LC-MS) run was completed and spectra were obtained, the spectra were searched against the *T. gondii* proteome database, v8 (http://www.toxodb.org/toxo/) using SEQUEST in Proteome Discoverer 1.4 (Thermo Electron). The search parameters permitted a 20-ppm precursor MS tolerance and a 1.0-Da MS/MS tolerance. Carbamidomethylation of cysteines was set as fixed modifications, and oxidation of methionine (M), phosphorylation on serine (S), threonine (T) and tyrosine (Y), SILAC labels of ^13^C_6_^15^N_2_ at lysine and of ^13^C_6_^15^N_4_ at arginine were allowed as variable modifications. Up to three missed tryptic cleavages of peptides were considered, and the false-discovery rate was set at 1% at the peptide level. The relative abundances of heavy-labeled (GST-D3-treated) and light-labeled (GST-treated) peptides (expressed as H/L ratios) were quantified by integrating the intensities of peptide ion elution profiles of the isotopologues with the Precursor Ions Quantifier node, and the probability of phosphorylation for each S/T/Y site on each peptide was calculated by the phosphoRS 3.0 node in Proteome Discoverer 1.4.

### (iv) Parallel reaction monitoring.

Parallel reaction monitoring (PRM) was carried out on the Q-Exactive mass spectrometer coupled to an EASY-nLC system (Thermo Fisher). Peptides were separated on a fused silica capillary (100 µm by 120 mm) packed with Halo C_18_ (2.7-µm particle size, 90-nm pore size, Michrom Bioresources) at a flow rate of 300 nl/min. Peptides were introduced into the mass spectrometer via a nanospray ionization source at a spray voltage of 2.0 kV. Mass spectrometry data were acquired with alternating MS-selected ion monitoring (SIM) scans and PRM (two scan groups), and lock mass function was activated (*m/z*, 371.1012; use lock masses, best; lock mass injection, full MS). Full scans were acquired from *m/z* 300 to 2,000 at 70,000 resolution (automatic gain control [AGC] target, 1e^6^; maximum ion time [max IT], 100 ms; profile mode). PRM was carried out with higher-energy collisional dissociation (HCD) MS/MS scans at 17,500 resolution on the precursors of interest (their *m/z* values were preimported into the inclusion list), with the following settings: AGC target, 5e^4^; max IT, 100 ms; isolation width of 1.6 *m/z*; and a normalized collisional energy of 35%. The light- and heavy-labeled TgAMA1 peptides were monitored with various charge states. The raw data were searched against the *T. gondii* proteome database, and the search files (.msf) imported into Skyline for selecting the precursor or transitions for quantitation. The raw data were exported from XCalibur to GraphPad Prism 6 for chromatogram plotting.

### Laser scanning cytometer-based invasion assay.

The two-color invasion assay was performed as previously described (56) with the following modifications. First, 3 × 10^6^ parasites were used to infect confluent HFF cells on 25-mm circular coverslips. The parasites were allowed to settle for 20 min at 21°C and then incubated for 1 h at 37°C. After fixation, samples were blocked for 1 h in PBS containing 2% BSA. The assays were performed 9 or 10 times, and each biological replicate was performed in duplicate.

### Heterologous TgAMA1 cleavage assay.

Human HEK293T cells were grown at 37°C and 5% CO_2_ in DMEM supplemented with 10 mM HEPES (pH 7.2), 2 mM l-glutamine, and 10% FBS and transfected with XtremeGENE HP DNA transfection reagent (Roche) as described previously (35). Transfected cells were pretreated for 5 min with DMEM containing 10 µM brefeldin A, followed by conditioning in DMEM in the presence of 10 µM brefeldin A and 5 µM GST or GST-D3 for 2.5 h. Cleaved GFP-TgAMA1 released into the culture supernatant was then detected by quantitative anti-green fluorescent protein (anti-GFP) Western blot analysis using the Odyssey imager.

## SUPPLEMENTAL MATERIAL

Figure S1  Allelic replacement by double homologous recombination at the *TgAMA1* locus. (A) Schematic of integration of the pA/TgAMA1^WT^ Flag.BLE vector at the *TgAMA1* genomic locus via homologous 5′- and 3′-flanking sequences. After allelic replacement, the bleomycin cassette (Ble) renders the parasites resistant to phleomycin. The primer pairs used to verify integration at the *TgAMA1* locus (p1 to p4) and the expected product sizes before and after allelic replacement are indicated. (B) Diagnostic PCR on cloned ARAMA1^WT^ parasites to confirm insertion of wild-type FLAG-tagged TgAMA1 at the endogenous *TgAMA1* locus. Blue dotted lines indicate PCR with primers p1 and p2, which gives the predicted 0.7-kb product after allelic replacement and 2-kb product in the parental Δ*ku80* Δ*HXG* parasite line. Red dotted lines indicate PCR with primers p3 and p4, which gives a 2.5-kb product after allelic replacement and a 1.5-kb product in the parental Δ*ku80* Δ*HXG* parasite line. (C) Immunofluorescence analysis of allelic replacement parasites expressing FLAG-tagged wild-type TgAMA1 (green), confirming the normal apical localization pattern of the introduced protein. Anti-TgIMC1 was used to stain the inner membrane complex (red). Bar = 5 µm. Download Figure S1, TIF file, 0.6 MB

Figure S2  GST-D3 reduces ionomycin-induced shedding of TgAMA1. ARAMA1^WT^ parasites were treated for the indicated time at 37°C with 1 µM ionomycin or without ionomycin and with either 1 µM GST-D3 (+) or GST (−). The pellet and supernatant fractions were analyzed by Western blotting using antibodies to TgMIC2 and TgAMA1. TgMLC1 was used as a loading control. Treatment with GST-D3 reduced both the constitutive (-ionomycin) and induced (+ionomycin) shedding of TgAMA1 into the supernatant, with no detected effect on the shedding of TgMIC2. Download Figure S2, TIF file, 0.3 MB

Figure S3  Treatment of parasites with TgRON2-2 causes a dose-dependent reduction in shedding of TgAMA1. (A) Western blot from microneme secretion assay comparing untreated ARAMA1^WT^ parasites (control) to parasites treated with 2.5 μM TgRON2-2. Assay pellet (P) and supernatant (S) fractions are indicated. TgMLC1 was used as a loading control. Arrowheads indicate full-length proteins (Full) and the corresponding shed ectodomains (Ecto). (B) Signal intensity ratio of ectodomain in the supernatant to full-length protein in the pellet from three independent microneme secretion assays (each represented by different color symbols) reveals a significant reduction in shedding of TgAMA1 in parasites treated with 2.5 μM TgRON2-2 compared to control parasites. Bars represent means with SEM. **, *P* = 0.0024 determined using a paired one-tailed *t* test. (C) Titration of the effect of TgRON2-2. Blots were probed for TgAMA1 (anti-FLAG) and TgMIC2. TgMLC1 was used as a loading control. (D) Quantification of the Western blots in panel C. The signal intensity ratio of shed ectodomain in the supernatant to full-length protein in the pellet was plotted for both TgAMA1 and TgMIC2. Download Figure S3, TIF file, 0.6 MB

Figure S4  The shedding of TgMIC8 is not affected by TgRON2-2 treatment. (A) Western blot from a microneme secretion assay comparing ARAMA1^WT^ parasites treated with 2.5 µM TgRON2-2 or without TgRON2-2. TgMLC1 was used as a loading control. Full, full-length TgMIC8; Ecto, ectodomain; P, pellet fraction; S, supernatant. (B) Quantification of the results from three independent secretion assays, showing signal intensity ratio of TgMIC8 ectodomain in the supernatant to full-length protein in the pellet. Paired signal intensity values from each biological replicate are indicated by the same color symbols. Bars indicate means with SEM. There was no significant (ns) change in the shedding of TgMIC8 upon treatment with TgRON2-2 (*P* = 0.2866 by paired one-tailed *t* test). Download Figure S4, TIF file, 0.2 MB

Figure S5  Dose dependence of the effect of GST-D3 on surface accumulation of TgAMA1 measured by flow cytometry. (A) ARAMA1^WT^ parasites were incubated with either GST (1 µM) or GST-D3 (0.0001 to 1 µM), and the amount of TgAMA1 on the parasite surface was determined by flow cytometry. Scatterplots of parasites treated with each concentration of GST-D3 (orange) were superimposed over the scatterplot of GST-treated parasites (purple). Alexa Fluor 546-FLAG TgAMA1 fluorescence is shown on the *x* axis, and Alexa Fluor 488-GST (peptide) fluorescence is shown on the *y* axis. (B) Percentage of parasites that were both GST and FLAG positive relative to the total number of parasites. Download Figure S5, TIF file, 1.4 MB

Figure S6  GST-D3 treatment does not increase the amount of TgSAG1 on the parasite surface. (Left) Representative histograms showing the median fluorescence intensity measured by flow cytometry of surface TgSAG1 in ARAMA^WT^ parasites treated with either 1 µM GST (purple) or GST-D3 (orange). Unstained parasites (dotted line) were used as gating controls. (Right) Combined flow data from five biological replicates. The median values of the TgAMA1 signals are indicated by bars. Paired signal intensity values from each replicate are plotted using the same color. GST-D3 treatment causes no significant (ns) accumulation of TgSAG1 (*P* = 0.6667) on the parasite surface as determined using a nonparametric two-tailed *t* test. Download Figure S6, TIF file, 0.2 MB

Figure S7  Identification of the TgAMA1 S527-containing phosphopeptide. The phosphopeptide GVQAAHHEHEFQS(phos)DR was identified via a SEQUEST search engine embedded in Proteome Discoverer 1.4 (Thermo Fisher) with an XCorr of 2.73, and precursor change in mass (ΔM) (between experimental *m*/*z* and theoretical *m/z*) of −2.3 ppm. (A) MS/MS spectrum of GVQAAHHEHEFQS(phos)DR. The relative intensities of *m/z* regions flanking the [parent+2H-98] were amplified 20 times to show the low-abundance *b* and *y* ions. The spectrum was manually annotated according to the Proteome Discoverer search results. (B) MS/MS spectrum annotated by Scaffold. The search result file (.msf) was imported into Scaffold 4.3 (Proteome software) for sequence annotation. Download Figure S7, TIF file, 0.5 MB

Figure S8  Quantification of the TgAMA1 S527-containing phosphopeptide from an independent biological replicate. Quantifications of TgAMA1 peptides were performed with precursor ion quantification and parallel reaction monitoring (PRM). The raw data were imported into Skyline for selecting the precursor or transitions for quantitation (MS1). As in Fig. 5, the phosphorylated form of GVQAAHHEHEFQSDR was found to be decreased in heavy-labeled (GST-D3-treated) parasites compared to light-labeled (GST-treated) parasites (H/L ratio of 0.49). In contrast, the nonphosphorylated TgAMA1 S527-containing peptide was relatively unchanged (H/L ratio of 0.97). The mean MS1 H/L ratio of the other TgAMA1 peptides (GVQAAHHEHEFQSDR, CLDYTELTDTVIER, NYGFYYVDTTGEGK, HLELQQPDRPPYR, and SVTENHHLIYGSAYVGENPDAFISK) in this experiment (such as NYGFYYVDTTGEGK shown here) was 0.98 ± 0.12 (PRM). PRM was simultaneously carried out in the same workflow as described in Materials and Methods. The mean H/L ratio of the phosphorylated form of GVQAAHHEHEFQSDR was 0.70 ± 0.041, which was calculated from the H/L ratios of the four transitions shown (precursor → R[y1], H/L ratio of 0.69; precursor → F[y5-98], H/L ratio of 0.69; precursor → V[b2], H/L ratio of 0.77; precursor → H[y10-98], H/L ratio of 0.66). Only one transition is shown for NYGFYYVDTTGEGK and the nonphosphorylated TgAMA1 S527-containing peptide. Download Figure S8, TIF file, 0.6 MB

Figure S9  Generation of parasites expressing TgAMA1 S527A or S527D and D558A mutations by allelic replacement. (A) Diagnostic PCR on individual parasite clones with FLAG-tagged AMA1^WT^, AMA1^S527A^, or AMA1^S527D^ at the endogenous *TgAMA1* locus. Blue dotted lines indicate PCR with primers p1 and p2, and red dotted lines indicate PCR with primer pair p3 and p4 (see Fig. S1 for the locations of primers and expected product sizes). Negative control indicates sample without the template. (B) Western blot comparing FLAG-TgAMA1 expression levels in the ARAMA1^WT^, ARAMA1^S527A^, ARAMA1^S527D^, and ARAMA1^D558A^ allelic replacement parasite lines. TgMLC1 was used as a loading control. (C) Anti-FLAG (green) immunofluorescence analysis of ARAMA1^S527A^, ARAMA1^S527D^, and ARAMA1^D558A^ allelic replacement parasite lines confirmed the proper apical localization of the mutant proteins. Anti-TgIMC1 (red) was used to stain the inner membrane complex. Bars = 5 µm. Download Figure S9, TIF file, 0.5 MB

Table S1  Primers used in this study. Table S1, DOCX file, 0.01 MB
