# Supplementary figures and images for: Not a Simple Tether: Binding of Toxoplasma gondii AMA1 to RON2 during Invasion Protects AMA1 from Rhomboid-Mediated Cleavage and Leads to Dephosphorylation of Its Cytosolic Tail
Source: mBio. 2016 Sep 13;7(5):e00754-16. doi: 10.1128/mBio.00754-16 (PMC5021801; doi:10.1128/mBio.00754-16)

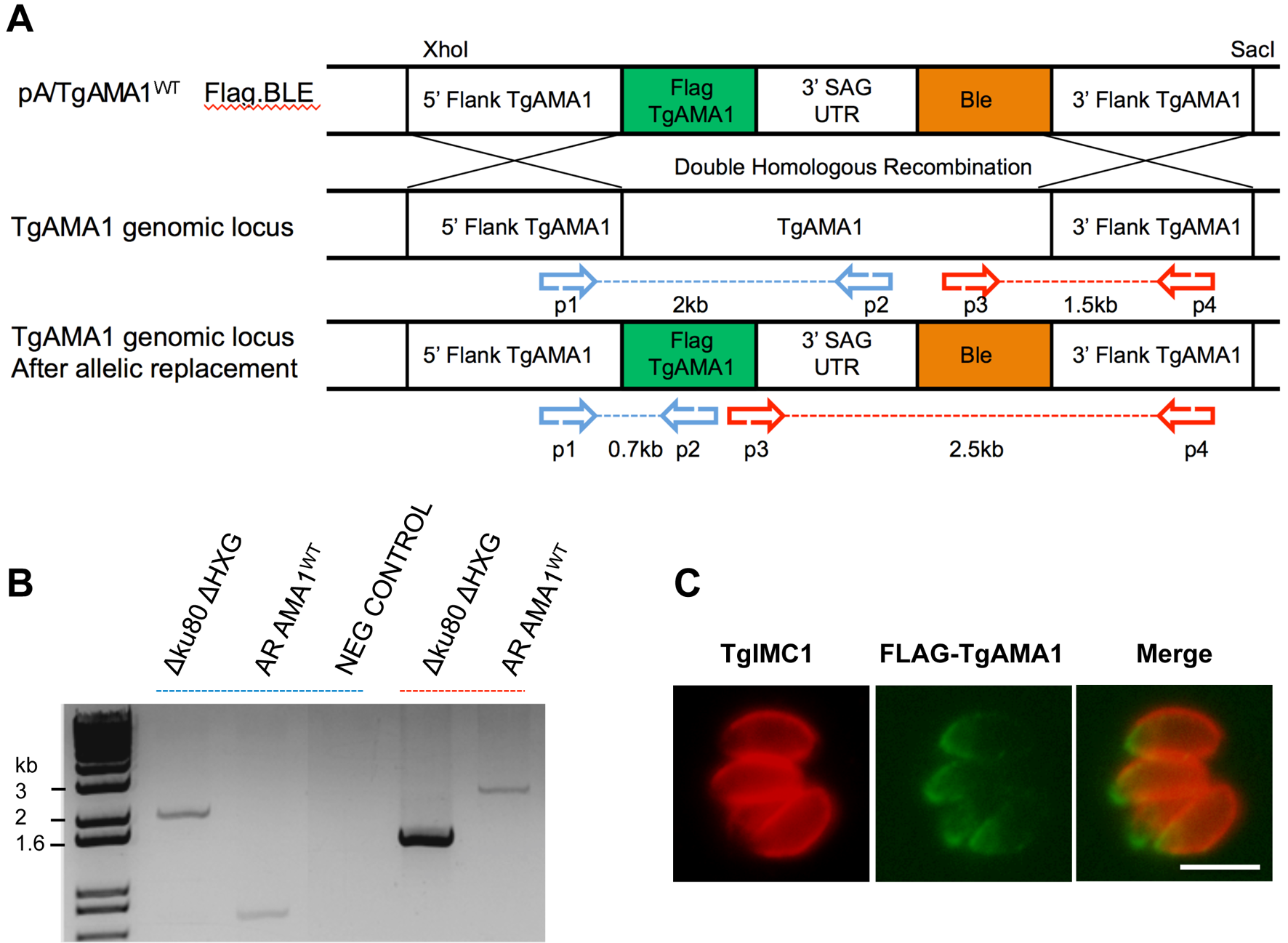

Supplement: Figure S1 — Allelic replacement by double homologous recombination at the TgAMA1 locus. (A) Schematic of integration of the pA/TgAMA1WT Flag.BLE vector at the TgAMA1 genomic locus via homologous 5′- and 3′-flanking sequences. After allelic replacement, the bleomycin cassette (Ble) renders the parasites resistant to phleomycin. The primer pairs used to verify integration at the TgAMA1 locus (p1 to p4) and the expected product sizes before and after allelic replacement are indicated. (B) Diagnostic PCR on cloned ARAMA1WT parasites to confirm insertion of wild-type FLAG-tagged TgAMA1 at the endogenous TgAMA1 locus. Blue dotted lines indicate PCR with primers p1 and p2, which gives the predicted 0.7-kb product after allelic replacement and 2-kb product in the parental Δku80 ΔHXG parasite line. Red dotted lines indicate PCR with primers p3 and p4, which gives a 2.5-kb product after allelic replacement and a 1.5-kb product in the parental Δku80 ΔHXG parasite line. (C) Immunofluorescence analysis of allelic replacement parasites expressing FLAG-tagged wild-type TgAMA1 (green), confirming the normal apical localization pattern of the introduced protein. Anti-TgIMC1 was used to stain the inner membrane complex (red). Bar = 5 µm. Download [file mbo004162990sf1.tif]

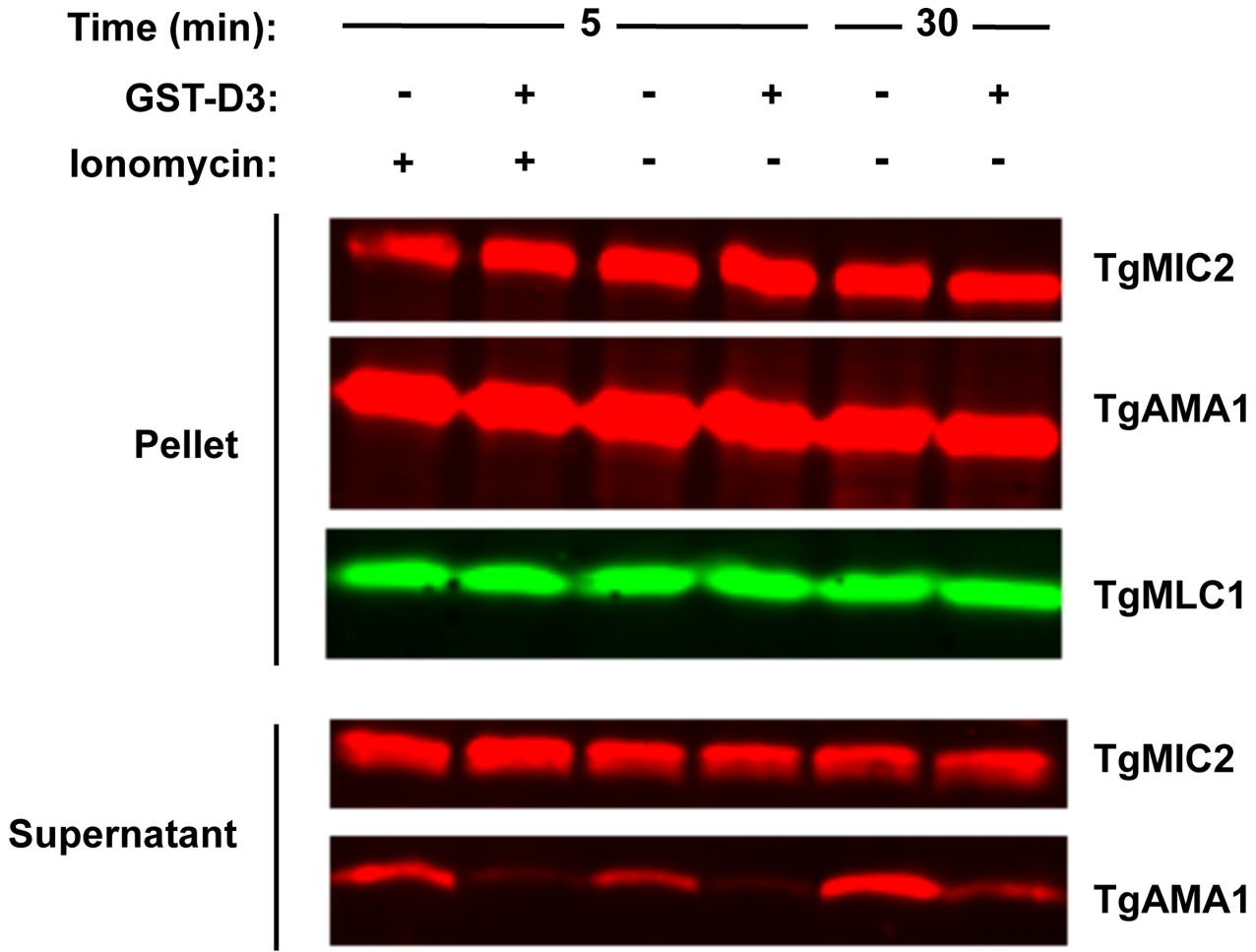

Supplement: Figure S2 — GST-D3 reduces ionomycin-induced shedding of TgAMA1. ARAMA1WT parasites were treated for the indicated time at 37°C with 1 µM ionomycin or without ionomycin and with either 1 µM GST-D3 (+) or GST (−). The pellet and supernatant fractions were analyzed by Western blotting using antibodies to TgMIC2 and TgAMA1. TgMLC1 was used as a loading control. Treatment with GST-D3 reduced both the constitutive (-ionomycin) and induced (+ionomycin) shedding of TgAMA1 into the supernatant, with no detected effect on the shedding of TgMIC2. Download [file mbo004162990sf2.tif]

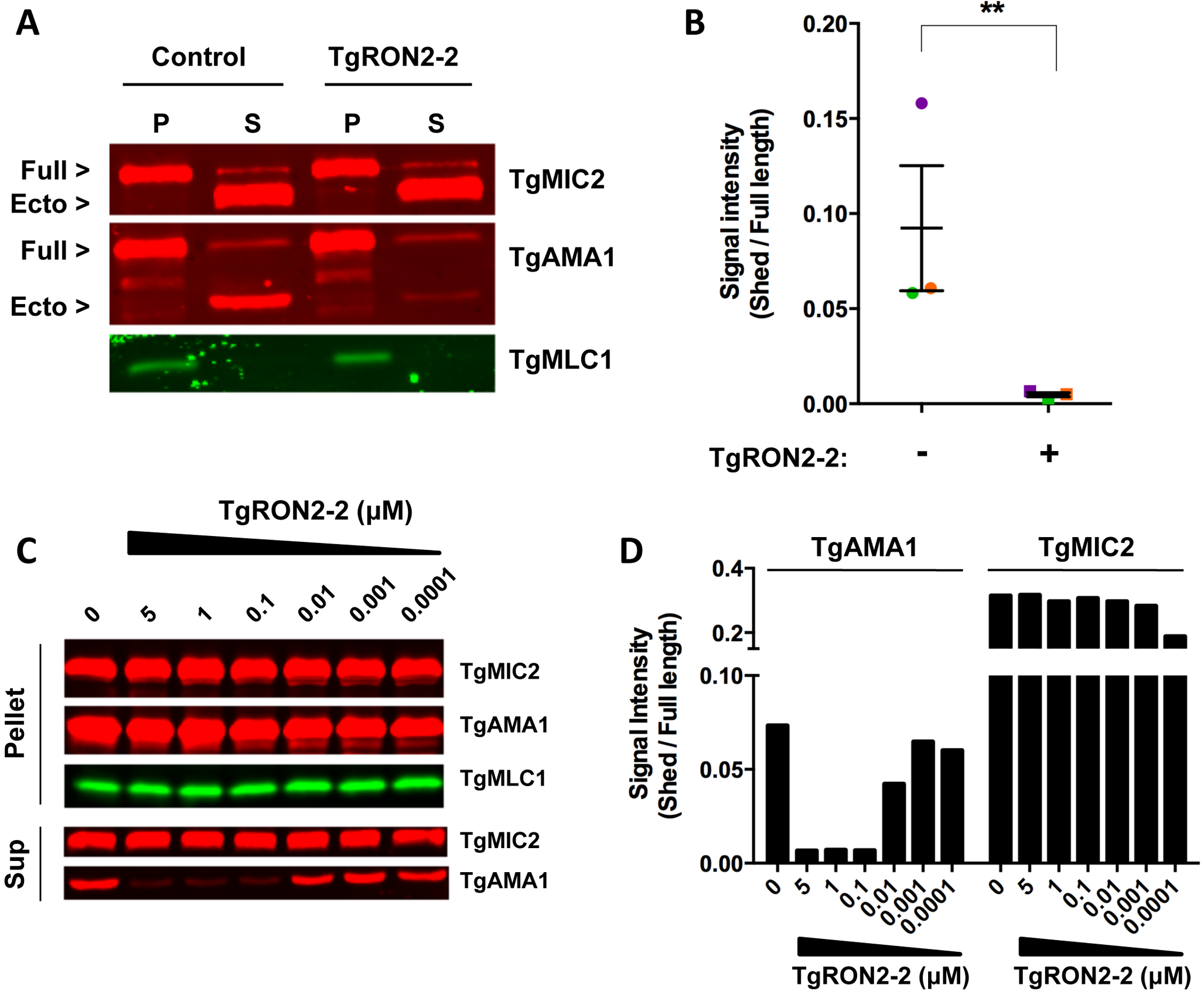

Supplement: Figure S3 — Treatment of parasites with TgRON2-2 causes a dose-dependent reduction in shedding of TgAMA1. (A) Western blot from microneme secretion assay comparing untreated ARAMA1WT parasites (control) to parasites treated with 2.5 μM TgRON2-2. Assay pellet (P) and supernatant (S) fractions are indicated. TgMLC1 was used as a loading control. Arrowheads indicate full-length proteins (Full) and the corresponding shed ectodomains (Ecto). (B) Signal intensity ratio of ectodomain in the supernatant to full-length protein in the pellet from three independent microneme secretion assays (each represented by different color symbols) reveals a significant reduction in shedding of TgAMA1 in parasites treated with 2.5 μM TgRON2-2 compared to control parasites. Bars represent means with SEM. **, P = 0.0024 determined using a paired one-tailed t test. (C) Titration of the effect of TgRON2-2. Blots were probed for TgAMA1 (anti-FLAG) and TgMIC2. TgMLC1 was used as a loading control. (D) Quantification of the Western blots in panel C. The signal intensity ratio of shed ectodomain in the supernatant to full-length protein in the pellet was plotted for both TgAMA1 and TgMIC2. Download [file mbo004162990sf3.tif]

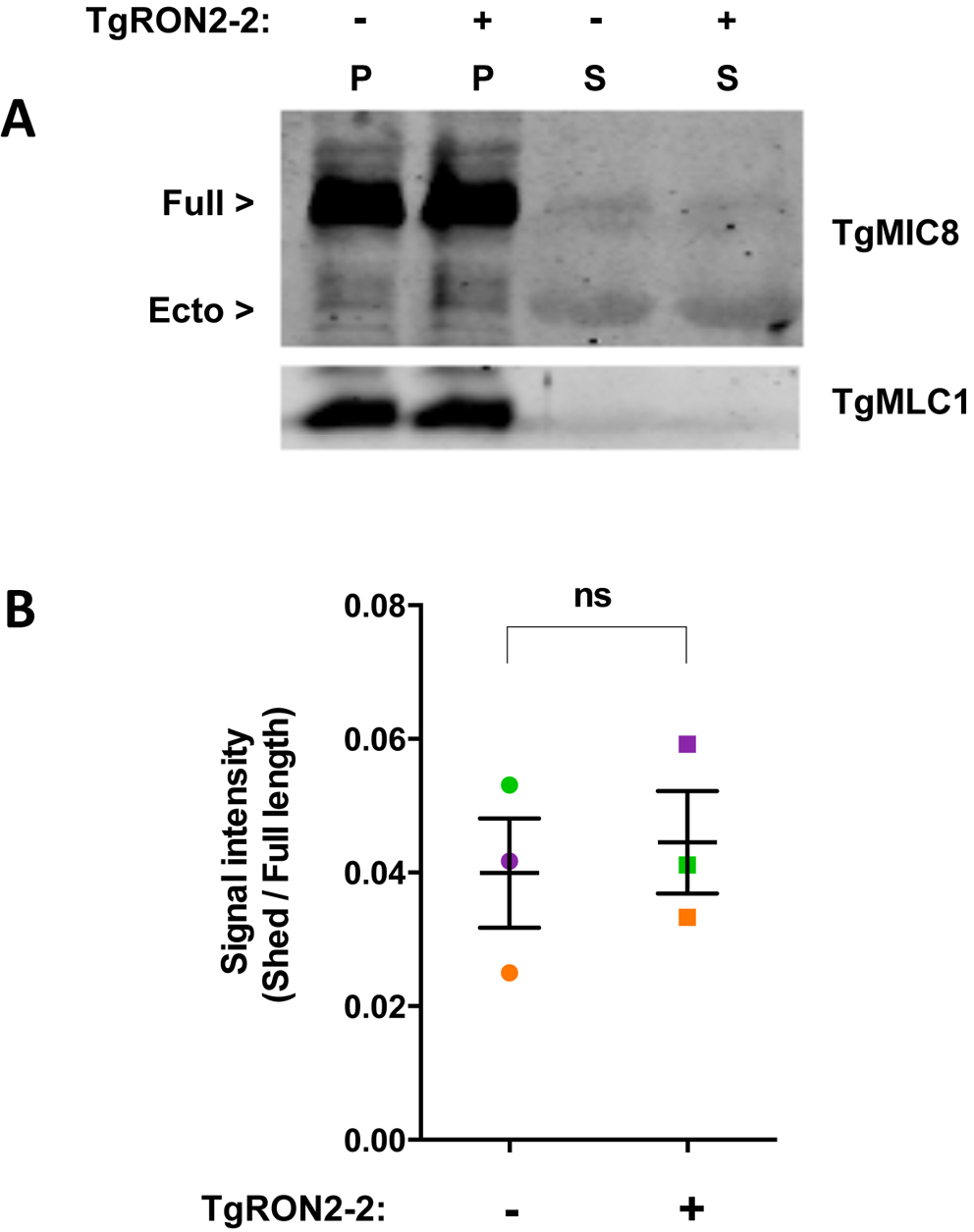

Supplement: Figure S4 — The shedding of TgMIC8 is not affected by TgRON2-2 treatment. (A) Western blot from a microneme secretion assay comparing ARAMA1WT parasites treated with 2.5 µM TgRON2-2 or without TgRON2-2. TgMLC1 was used as a loading control. Full, full-length TgMIC8; Ecto, ectodomain; P, pellet fraction; S, supernatant. (B) Quantification of the results from three independent secretion assays, showing signal intensity ratio of TgMIC8 ectodomain in the supernatant to full-length protein in the pellet. Paired signal intensity values from each biological replicate are indicated by the same color symbols. Bars indicate means with SEM. There was no significant (ns) change in the shedding of TgMIC8 upon treatment with TgRON2-2 (P = 0.2866 by paired one-tailed t test). Download [file mbo004162990sf4.tif]

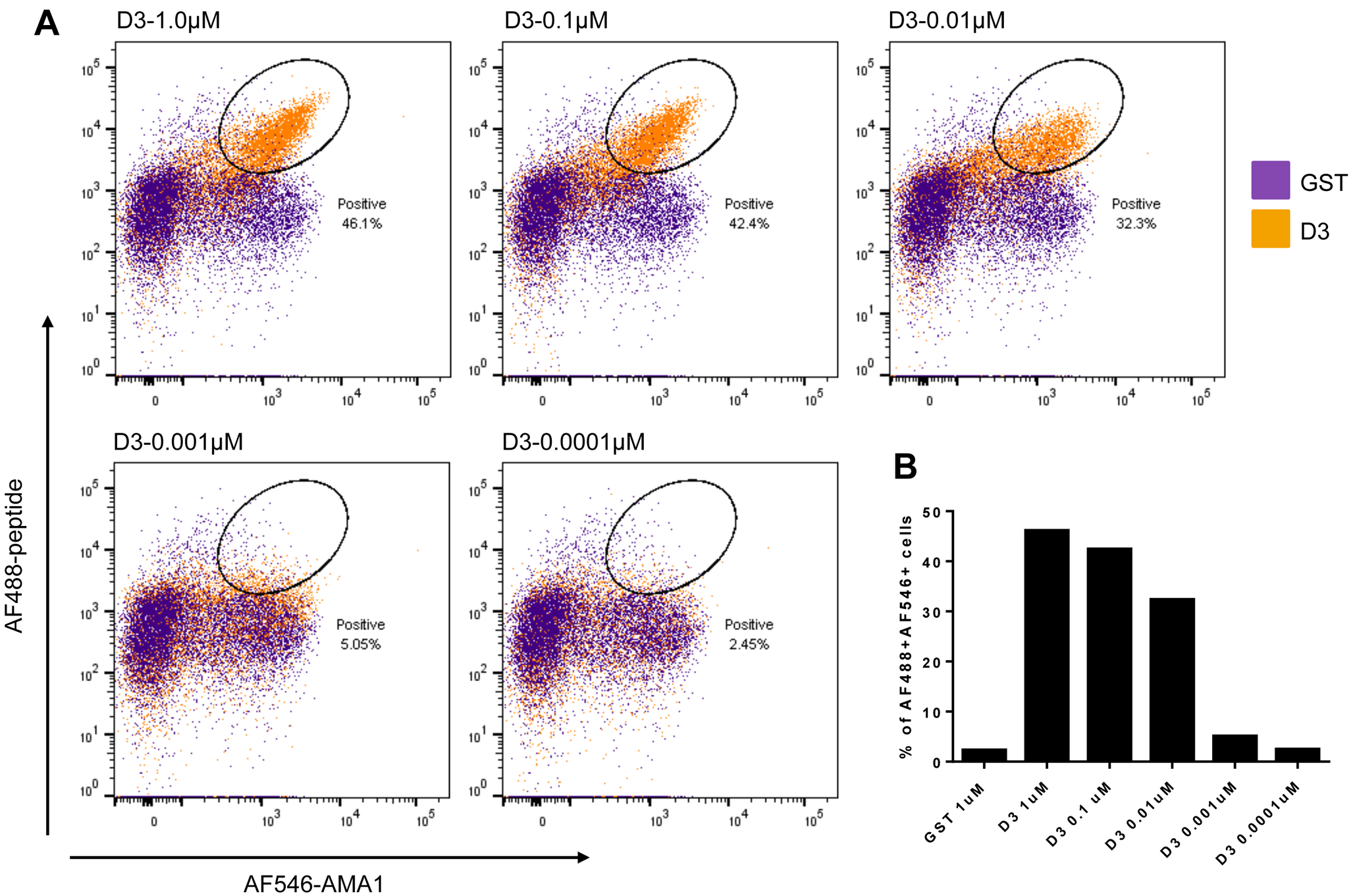

Supplement: Figure S5 — Dose dependence of the effect of GST-D3 on surface accumulation of TgAMA1 measured by flow cytometry. (A) ARAMA1WT parasites were incubated with either GST (1 µM) or GST-D3 (0.0001 to 1 µM), and the amount of TgAMA1 on the parasite surface was determined by flow cytometry. Scatterplots of parasites treated with each concentration of GST-D3 (orange) were superimposed over the scatterplot of GST-treated parasites (purple). Alexa Fluor 546-FLAG TgAMA1 fluorescence is shown on the x axis, and Alexa Fluor 488-GST (peptide) fluorescence is shown on the y axis. (B) Percentage of parasites that were both GST and FLAG positive relative to the total number of parasites. Download [file mbo004162990sf5.tif]

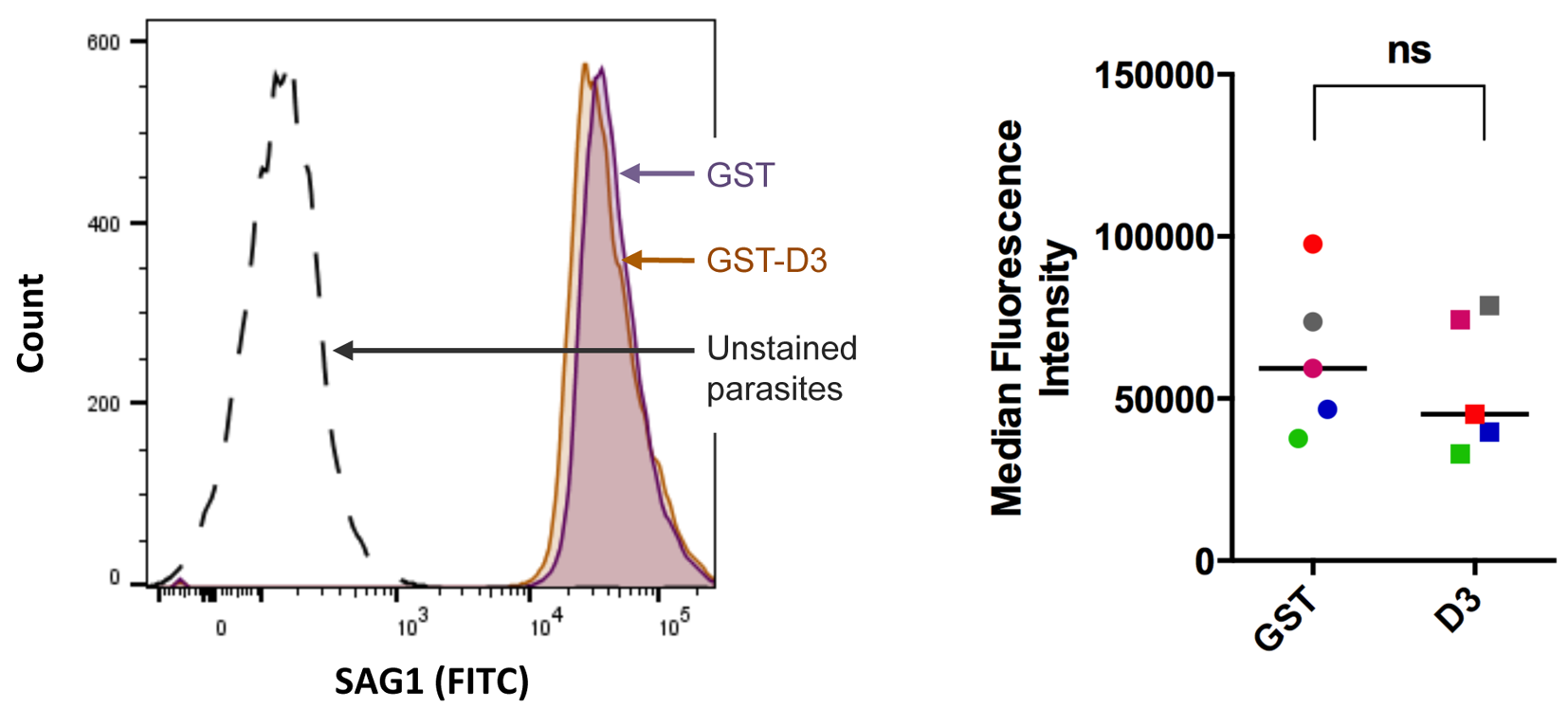

Supplement: Figure S6 — GST-D3 treatment does not increase the amount of TgSAG1 on the parasite surface. (Left) Representative histograms showing the median fluorescence intensity measured by flow cytometry of surface TgSAG1 in ARAMAWT parasites treated with either 1 µM GST (purple) or GST-D3 (orange). Unstained parasites (dotted line) were used as gating controls. (Right) Combined flow data from five biological replicates. The median values of the TgAMA1 signals are indicated by bars. Paired signal intensity values from each replicate are plotted using the same color. GST-D3 treatment causes no significant (ns) accumulation of TgSAG1 (P = 0.6667) on the parasite surface as determined using a nonparametric two-tailed t test. Download [file mbo004162990sf6.tif]

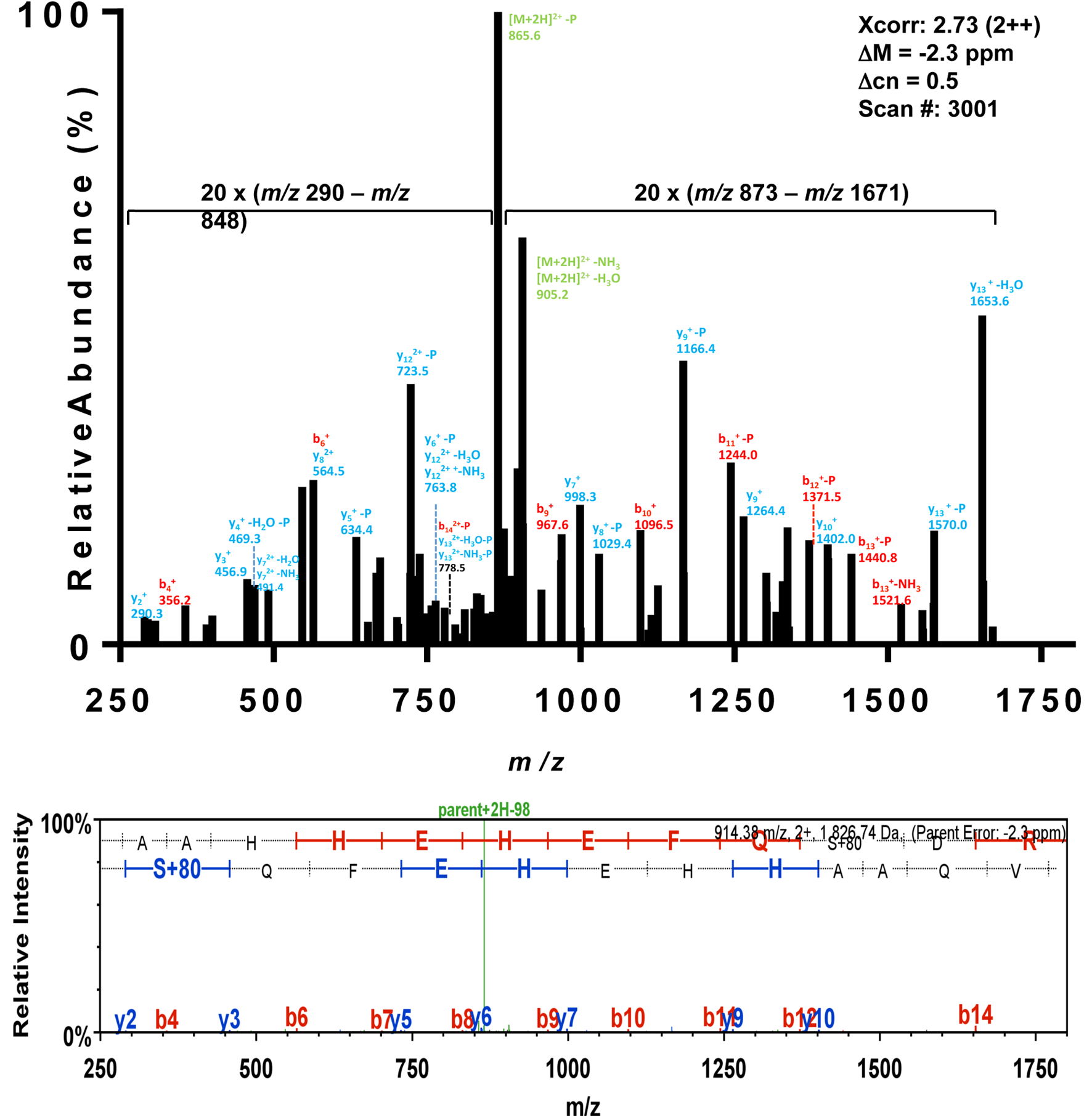

Supplement: Figure S7 — Identification of the TgAMA1 S527-containing phosphopeptide. The phosphopeptide GVQAAHHEHEFQS(phos)DR was identified via a SEQUEST search engine embedded in Proteome Discoverer 1.4 (Thermo Fisher) with an XCorr of 2.73, and precursor change in mass (ΔM) (between experimental m/z and theoretical m/z) of −2.3 ppm. (A) MS/MS spectrum of GVQAAHHEHEFQS(phos)DR. The relative intensities of m/z regions flanking the [parent+2H-98] were amplified 20 times to show the low-abundance b and y ions. The spectrum was manually annotated according to the Proteome Discoverer search results. (B) MS/MS spectrum annotated by Scaffold. The search result file (.msf) was imported into Scaffold 4.3 (Proteome software) for sequence annotation. Download [file mbo004162990sf7.tif]

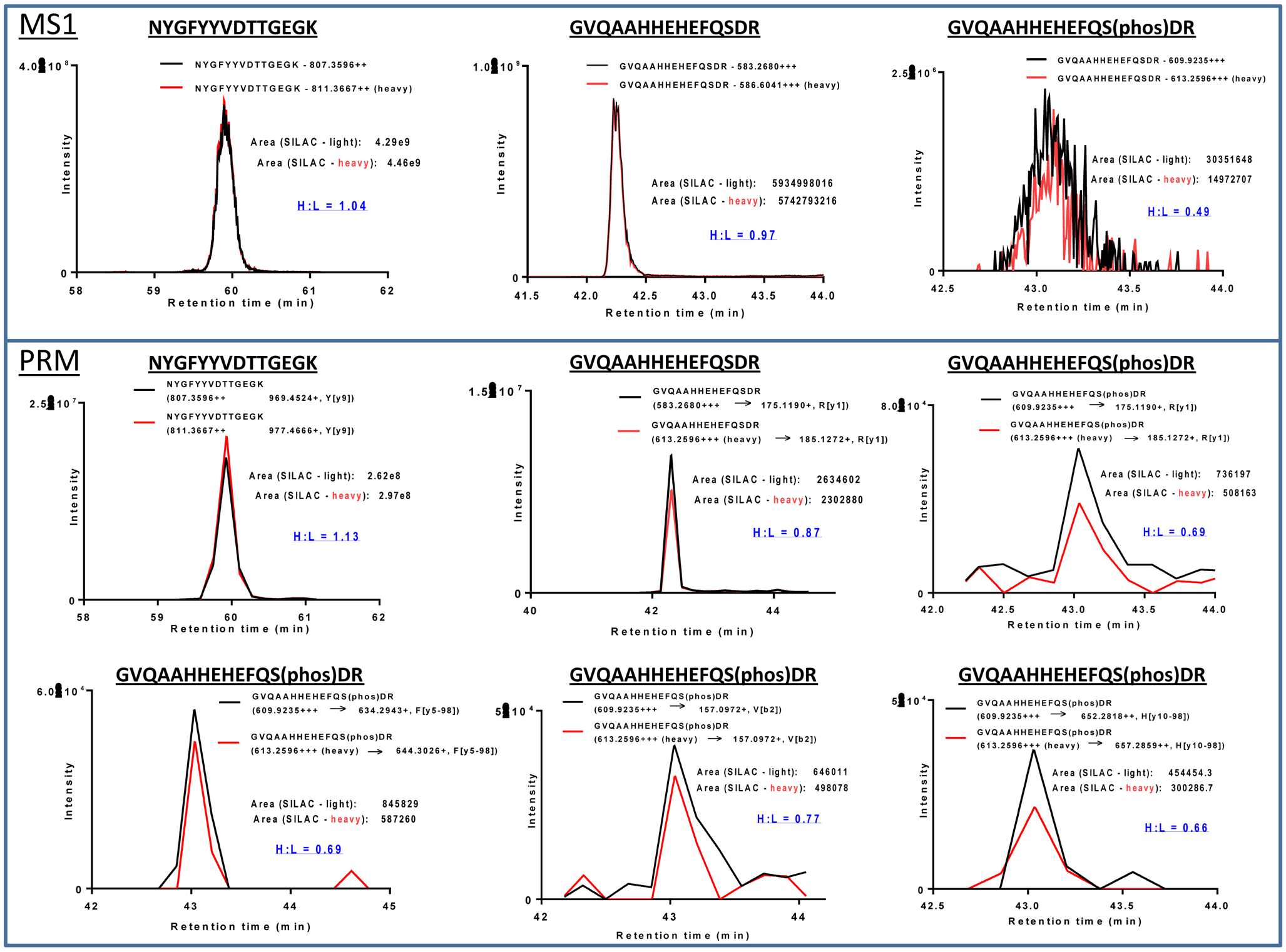

Supplement: Figure S8 — Quantification of the TgAMA1 S527-containing phosphopeptide from an independent biological replicate. Quantifications of TgAMA1 peptides were performed with precursor ion quantification and parallel reaction monitoring (PRM). The raw data were imported into Skyline for selecting the precursor or transitions for quantitation (MS1). As in Fig. 5, the phosphorylated form of GVQAAHHEHEFQSDR was found to be decreased in heavy-labeled (GST-D3-treated) parasites compared to light-labeled (GST-treated) parasites (H/L ratio of 0.49). In contrast, the nonphosphorylated TgAMA1 S527-containing peptide was relatively unchanged (H/L ratio of 0.97). The mean MS1 H/L ratio of the other TgAMA1 peptides (GVQAAHHEHEFQSDR, CLDYTELTDTVIER, NYGFYYVDTTGEGK, HLELQQPDRPPYR, and SVTENHHLIYGSAYVGENPDAFISK) in this experiment (such as NYGFYYVDTTGEGK shown here) was 0.98 ± 0.12 (PRM). PRM was simultaneously carried out in the same workflow as described in Materials and Methods. The mean H/L ratio of the phosphorylated form of GVQAAHHEHEFQSDR was 0.70 ± 0.041, which was calculated from the H/L ratios of the four transitions shown (precursor → R[y1], H/L ratio of 0.69; precursor → F[y5-98], H/L ratio of 0.69; precursor → V[b2], H/L ratio of 0.77; precursor → H[y10-98], H/L ratio of 0.66). Only one transition is shown for NYGFYYVDTTGEGK and the nonphosphorylated TgAMA1 S527-containing peptide. Download [file mbo004162990sf8.tif]

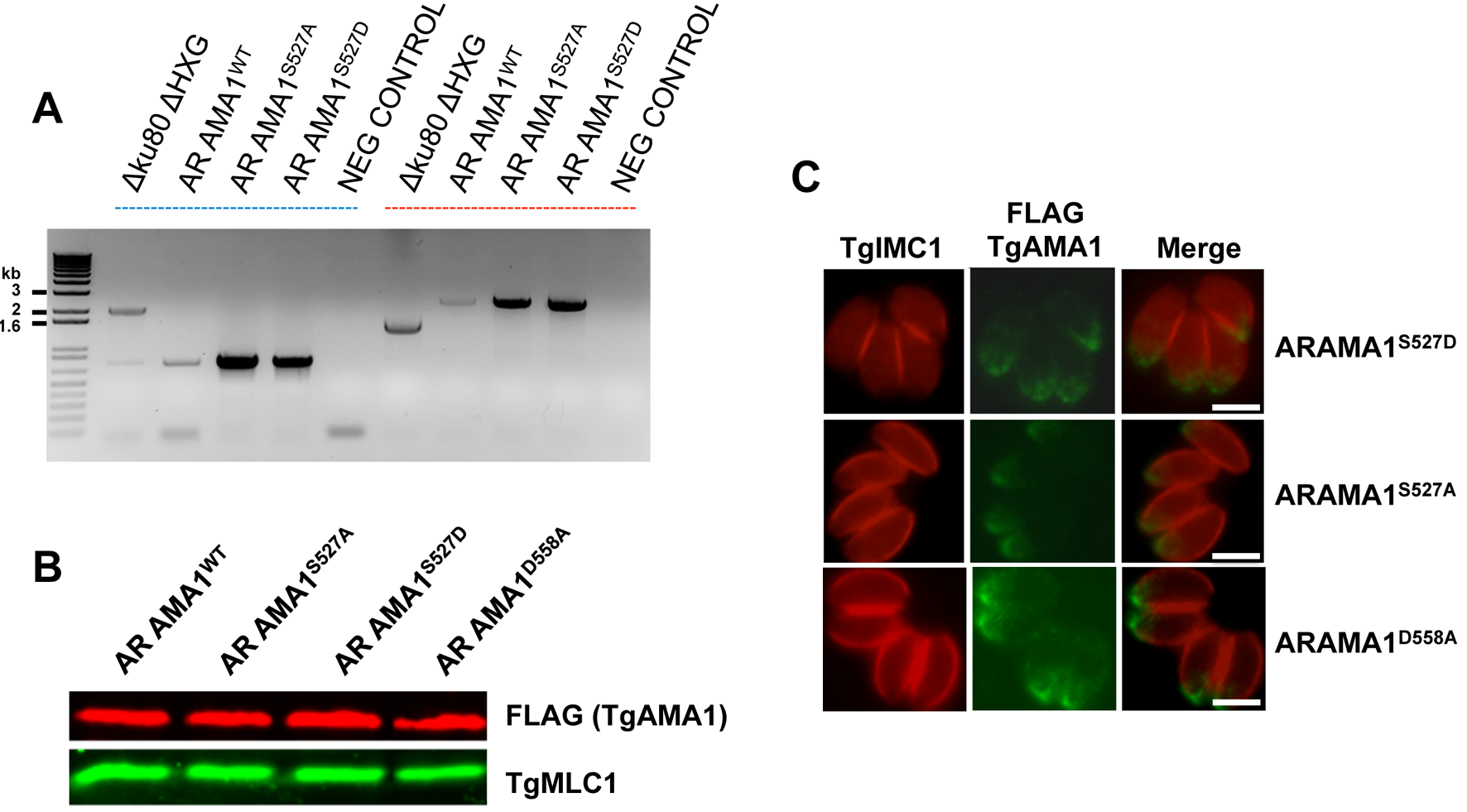

Supplement: Figure S9 — Generation of parasites expressing TgAMA1 S527A or S527D and D558A mutations by allelic replacement. (A) Diagnostic PCR on individual parasite clones with FLAG-tagged AMA1WT, AMA1S527A, or AMA1S527D at the endogenous TgAMA1 locus. Blue dotted lines indicate PCR with primers p1 and p2, and red dotted lines indicate PCR with primer pair p3 and p4 (see Fig. S1 for the locations of primers and expected product sizes). Negative control indicates sample without the template. (B) Western blot comparing FLAG-TgAMA1 expression levels in the ARAMA1WT, ARAMA1S527A, ARAMA1S527D, and ARAMA1D558A allelic replacement parasite lines. TgMLC1 was used as a loading control. (C) Anti-FLAG (green) immunofluorescence analysis of ARAMA1S527A, ARAMA1S527D, and ARAMA1D558A allelic replacement parasite lines confirmed the proper apical localization of the mutant proteins. Anti-TgIMC1 (red) was used to stain the inner membrane complex. Bars = 5 µm. Download [file mbo004162990sf9.tif]
