# Supplementary material for: Not a Simple Tether: Binding of Toxoplasma gondii AMA1 to RON2 during Invasion Protects AMA1 from Rhomboid-Mediated Cleavage and Leads to Dephosphorylation of Its Cytosolic Tail
Source: mBio. 2016 Sep 13;7(5):e00754-16. doi: 10.1128/mBio.00754-16 (PMC5021801; doi:10.1128/mBio.00754-16)
Supplement: Table S1 — Primers used in this study. [file mbo004162990st1.docx]

**Table S1: Primers used in this study**

| Primer name | Primer Sequence 5’ to 3’ |
| --- | --- |
| S527A fwd | atgaacatgagtttcaggcagacagaggtgctcga |
| S527A rev | tcgagcacctctgtctgcctgaaactcatgttcat |
| S527D fwd | ctcatcatgaacatgagtttcaggatgacagaggtgctcgaaaaaagag |
| S527D rev | Ctcttttttcgagcacctctgtcatcctgaaactcatgttcatgatga |
| D558A fwd | agaggagaacattgaacaagctggggaaacacatgttatg |
| D558A rev | cataacatgtgtttccccagcttgttcaatgttctcctct |
| TgAMA1KOZAK fwd (p1) | Cactgagacgaagcacatggggctcgtgg |
| TgAMA1EXON3 rev(p2) | Ggcatttactgatgaacgcatctgggttc |
| TgAMA1EXON7 fwd (p3) | Cgtccaggcggctcatcatg |
| TgAMA13’FLANK AR rev (p4) | aatgccactggtgtcccaacgtaac |
